# What happens after a blood meal? A transcriptome analysis of the main tissues involved in egg production in *Rhodnius prolixus*, an insect vector of Chagas disease

**DOI:** 10.1371/journal.pntd.0008516

**Published:** 2020-10-15

**Authors:** Jimena Leyria, Ian Orchard, Angela B. Lange

**Affiliations:** Department of Biology, University of Toronto Mississauga, Mississauga, ON, Canada; National Institute of Allergy and Infectious Diseases, UNITED STATES

## Abstract

The blood-sucking hemipteran *Rhodnius prolixus* is a vector of Chagas disease, one of the most neglected tropical diseases affecting several million people, mostly in Latin America. The blood meal is an event with a high epidemiological impact since adult mated females feed several times, with each meal resulting in a bout of egg laying, and thereby the production of hundreds of offspring. By means of RNA-Sequencing (RNA-Seq) we have examined how a blood meal influences mRNA expression in the central nervous system (CNS), fat body and ovaries in order to promote egg production, focusing on tissue-specific responses under controlled nutritional conditions. We illustrate the cross talk between reproduction and a) lipids, proteins and trehalose metabolism, b) neuropeptide and neurohormonal signaling, and c) the immune system. Overall, our molecular evaluation confirms and supports previous studies and provides an invaluable molecular resource for future investigations on different tissues involved in successful reproductive events. These analyses serve as a starting point for new investigations, increasing the chances of developing novel strategies for vector population control by translational research, with less impact on the environment and more specificity for a particular organism.

## Author summary

The blood-sucking hemipteran *Rhodnius prolixus* is one of the main vectors of Chagas disease. The blood meal is an event with a high epidemiological impact since adult mated females take several blood meals, with each meal resulting in the laying of a batch of eggs, thereby resulting in hundreds of offspring. This work describes an in-depth central nervous system (CNS), ovary and fat body transcriptome analysis, focusing on transcripts related to blood intake which may be relevant in promoting egg production. To date, the principle focus in Chagas disease prevention is on the elimination of triatomine vectors and their progeny. This work will serve as a starting point for initiating novel investigations on targets identified with a potential for use in vector control; for example, using specific genes to generated symbiont-mediated RNAi, a powerful technology which provides a novel means in biocontrol against tropical disease vectors.

## Introduction

Insects, which represent more than half of all living organisms on earth, have a close relationship with human beings. To many of them, we can ascribe a negative interaction, for example those that act as carriers of disease. Chagas disease, one of the most neglected tropical diseases, is caused by the protozoan *Trypanosoma cruzi*, which is transmitted to mammalian hosts primarily by blood-feeding insects, the triatomines [1]. This disease affects 6–7 million people, mostly in Latin America, but because of migration the disease has spread to other continents [2]. To date, treatment of the chronic phase of this disease is limited [3], resulting in 2000 deaths per year [1], although it is known that Chagas disease is an under-reported cause of death [4]. The principle focus in Chagas disease prevention is on the elimination of triatomine vectors from human homes. Currently, the most heavily used option is chemical control, although resistance to these insecticides has been reported in the last decade [5]. Furthermore, the devastating impact of chemical insecticides on the environment and other organisms, such as beneficial insects, can no longer be ignored [6].

Triatomines have developed an integrated control over the reproductive system, whereby different tissues work with extreme precision and coordination to achieve successful production of progeny. There are three tissues that work in concert to promote reproduction; the central nervous system (CNS), fat body and ovaries. The CNS contains neuroendocrine cells that synthesize neuropeptides involved in the coordination of events that promote egg production. These neuropeptides are produced as large precursors, which are then cleaved and modified to become biologically active neuropeptides [7]. These neuropeptides are secreted as neuromodulators or neurohormones to act via specific receptors [8]. With regard to reproduction, these receptors are located on the fat body and ovaries. The fat body is a multifunctional organ analogous to vertebrate adipose tissue and liver. It is considered an interchanging center, remotely integrating with the CNS to regulate physiology by sensing hormonal and nutritional signals and responding by mobilizing stored nutrients such as proteins, carbohydrates and lipids, for use in egg formation, or during periods of inactivity or nutritional shortage [9, 10]. Apart from these storage functions, the fat body is also involved in the regulation of hematopoiesis, innate immune homeostasis and detoxification [10].

In oviparous organisms, including triatomines, embryonic development occurs apart from the maternal body. Egg survival, therefore, depends on the utilization of previously stored material taken up by the oocytes, such as proteins, lipids, carbohydrates and other minor components, all of which are synthesized mainly by the fat body [11]. Insect oocytes are specific structures designed to select, internalize, and store nutrients, such as yolk granules and lipid droplets. The process of yolk deposition is termed vitellogenesis, which represents a phase of accelerated egg growth leading to the production of mature eggs in a relatively short period of time [11]. The CNS-fat body-ovary axis is essential for triatomines to produce viable eggs. Interestingly, the trigger for this interaction is a single blood meal. Although in some colonies of the triatomine, *Rhodnius prolixus*, unfed females can make a small number of eggs from resources that may remain after molting to an adult (autogeny) [12], the large batch of eggs is triggered by ingestion of a blood meal. After a blood meal, a *R*. *prolixus* female can produces up to 30 eggs during the following three weeks [13]. For this reason, knowledge of the molecular and cellular mechanisms used in egg formation are essential to develop novel strategies of vector population control.

In addition to being a main vector of Chagas disease, with high epidemiological relevance for easily colonizing domestic habitats [14], *R*. *prolixus* has been the subject of intense investigations over the past century, which have contributed to our understanding of important aspects of metabolism and physiology in insects [15]. It is important to highlight that the complete genome of *R*. *prolixus* has been published [16] and, therefore, many new questions can be asked and answered with regard to insect physiology/endocrinology. Next-generation sequencing allows us to study biological systems at the genomic level to link mRNA sequences with specific biological functions of specific tissues during a particular stage or state. Recently, by transcriptome analysis we reported an up-regulation of transcripts involved in insulin-like peptide/target of rapamycin (ILP/ToR) signaling in unfed insects. However, we demonstrated that this signaling pathway is only activated in the fat body and ovaries of fed insects. Thus, we demonstrated that unfed females are in a sensitized state to respond to an increase of ILP levels by rapidly activating ILP/ToR signaling after a blood meal [17]. Here, we examine how a blood meal influences CNS, fat body and ovary gene expression to promote egg production; focusing on details associated with tissue-specific responses in particular nutritional states. Our data opens up avenues for new investigations on targets identified with a potential for translational research to generate novel strategies of vector population control, with less impact in the environment and with more specificity for a particular organism. For example, targeting specific genes for symbiont-mediated RNAi; a powerful technology which provides a potential means in biocontrol against tropical disease vectors [18].

## Materials and methods

### Insects

Insects were maintained in an incubator at 25°C under high humidity (~50%). For the experiments, males and females were separated during the last nymphal instar (fifth stage) and 30 days post-ecdysis were fed on defibrinated rabbit blood (Cedarlane Laboratories Inc., Burlington, ON, Canada) through an artificial feeding membrane. Insects that gorged at least nine times their initial body weight (typical of complete gorging) were selected and allowed to molt into the adult stage. Newly-emerged adult females were segregated and placed together with a recently fed male to copulate. Then, females at 10 days post-ecdysis were offered a blood meal and only insects that fed 2.5 to 3 times their initial body weight (typical of adult gorging) were used for the experiments. Insects in the fed condition will have begun vitellogenesis and egg growth. CNS, fat body (FB) and ovaries (OV) from adult mated females were dissected at 10 days post-ecdysis for the unfed condition (UFC) and at 3 days post-feeding as the fed condition (FC), according to Leyria et al. [17]. OV_FC means follicles containing mature oocytes, while OV_UFC are complete ovarioles [17].

### Transcriptomic data analysis

Read sequences were obtained from Leyria et al. [17]. This study reported transcriptomes of CNS, FB and OV from fed and unfed females. The raw sequence dataset of this project is registered with the National Center for Biotechnology Information (NCBI) under PRJNA624187 and PRJNA624904 BioProjects. A detailed description of our bioinformatic pipeline can be found in Leyria et al. [17]. Briefly, CNS, OV and ventral and dorsal FB of *R*. *prolixus* females were dissected in cold autoclaved phosphate buffered saline (PBS, 6.6 mM Na_2_HPO_4_/KH_2_PO_4_, 150 mM NaCl, pH 7.4). Three independent experiments were analyzed (n = 3) with each n composed of tissues from 10 insects. RNA extraction was performed with Trizol reagent (Invitrogen by Thermo Fisher Scientific, MA, USA), followed by DNase treatment (Millipore-Sigma, WI, USA) and then repurified with PureLink RNA Mini Kit (Ambion by Thermo Fisher Scientific, MA, USA). Libraries for sequencing were made from high quality RNA that were generated using NEBNext Ultra RNA Library Prep Kit for Illumina (New England Biolabs, MA, USA) following manufacturer’s recommendations. The libraries were sequenced on Illumina HiSeq platforms (*HiSeq 2500*) at the Novogene sequencing facility (California, USA). Raw data were recorded in a FASTQ file, which contains sequence (reads) and corresponding sequencing quality information. Fastq format were first processed through in-house perl scripts, where clean data (clean reads) were obtained by removing reads containing the adapter, reads containing ploy-N and low quality reads from raw data. Also, Q20, Q30 and GC content from the clean data were calculated. All the downstream analyses were based on the clean data [17].

### Differential expression analysis

The data was analyzed using gene annotation from the RproC1.3 gene set (ftp://ftp.ensemblgenomes.org/pub/metazoa/release42/gff3/rhodnius_prolixus/Rhodnius_prolixus.RproC3.42.gff3.gz), and *R*. *prolixus* alternative annotation gene set [16]. First, clean reads were aligned to the reference genome using HISAT2 software. After that, HTSeq v0.6.1 was used to count the number of reads mapped to each gene. FPKM (expected number of Fragments Per Kilobase of transcript sequence per Millions base pairs sequenced) of each gene were calculated based on the length of the gene and number of reads mapped to the gene. In general, an FPKM value of 0.1 was set as the threshold for determining whether the gene is expressed or not. Differential expression analysis of two nutritional conditions were performed using the DESeq R package (1.18.0). DESeq provides statistical routines for determining differential expression in digital gene expression data using a model based on the negative binomial distribution. The resulting *P*-values were adjusted using the Benjamini and Hochberg’s approach for controlling the false discovery rate. Genes with an adjusted *P*-value < 0.05 found by DESeq were assigned as differentially expressed. We performed heatmap analysis to compare mRNA expression levels of FB, OV and/or CNS of female adults in different nutritional conditions. The input data was the readcount values obtained by gene expression analysis after normalization and is presented by means of a colour scale. All the numeric information of the heatmap charts are shown in several worksheets of S1 Table and S2 Table, including the fold changes in expression (log_2_(FC/UFC)). Gene Ontology (GO) enrichment analysis of differentially expressed genes was implemented by the GOseq R package, in which gene length bias was corrected. GO terms with corrected *P*-value less than 0.05 were considered significantly enriched by differentially expressed genes.

### Validation of RNA-Seq data

Reverse transcription quantitative real-time polymerase chain reaction (RT-qPCR) is a powerful tool for validating gene expression differences due to its sensitivity and specificity. As we mainly focused our attention on the FB and OV, 7 transcripts were chosen at random and their transcript expressions were analyzed by RT-qPCR on these tissues to validate differentially expressed genes obtained by Illumina sequencing: *trehalose transporter* (RPRC007957); *vitellogenin-1* (RPRC013511); *trehalose-6-phosphate synthase* (RPRC003010); *trehalase-1* (RPRC012647); *fatty acid synthase* (RPRC000269), *SREBP* (RPRC014734); and *lipophorin receptor* (RPRC011390). Briefly, total RNA was extracted as described above. The final concentration and A260/280 ratio of purified RNA were measured using the spectrophotometer DS-11+ (DeNovix Inc., Wilmington, DE, USA). All samples showed a ratio between 1.9 and 2.0. RNA integrity, including potential degradation products and DNA contamination, was evaluated by electrophoresis in a 1% agarose gel (FroggaBio Inc., Concord, ON, Canada). RNA was considered intact when the 18S rRNA band was observed. cDNAs were synthesized from 1 μg of total RNA by reverse transcription reaction using random primers and 50 U of MultiScribe MuLV reverse transcriptase (High Capacity cDNA Reverse Transcription Kit, Applied-Biosystems, by Fisher Scientific, ON, Canada). The conditions of the thermal cycler were: 10 min at 25°C, 120 min at 37°C, and 5 min at 85°C. The cDNAs obtained were diluted 10-fold for the experiments. qPCRs were performed using an advanced master mix with super green low rox reagent (Wisent Bioproducts Inc, QC, Canada), according to manufacturer's recommendations, using 4 pmol of sense and antisense primers in a final volume of 10 μl. The qPCR temperature-cycling profile was: initial denaturation 3 min at 95°C, followed by 39 cycles of 30 s at 94°C, 30 s at 58–60°C (depending on the pair of primers used), and 1 min at 72°C, followed by a final extension at 72°C for 10 min. Three independent experiments were performed (n = 3) with each n composed of tissues from 5 insects. Each reaction contained 3 technical replicates as well as a no template control (cDNA replaced by nuclease-free water, to identify set-up contamination and primer-dimer product amplification) and a no reverse transcriptase control (to confirm the effectiveness of the DNAse I treatment). qPCR was performed using a CFX384 Touch Real-Time PCR Detection System (BioRad Laboratories Ltd., Mississauga, ON, Canada). The sequences of primers used for amplification (by Sigma-Aldrich, ON, Canada) and the efficiencies which validate their use, are shown in the S3 Table. For each pair of primers a dissociation curve with a single peak was seen, indicating that a single cDNA product was amplified. β-actin, which was previously validated for transcript expression in FB and OV from *R*. *prolixus* at different nutritional conditions [17], was used as the reference gene. The results, i.e. Cq of each reaction, were analyzed by the 2^-ΔΔCt^ method [19]. Specific target amplification was confirmed by automated sequencing (Macrogen, NY, USA). The correlation coefficient between Illumina RNA sequencing and RT-qPCR data was analyzed by the Pearson’s test.

### Lipid and carbohydrate measurements

Ovaries and ventral and dorsal FB were dissected from insects during the UFC and FC under cold *R*. *prolixus* saline (150 mM NaCl, 6 mM KCl8, 2.0 mM CaCl_2_, 8.5 mM MgCl_2_, 4.0 mM NaHCO_3_, 5.0 mM HEPES, pH 7.0) [20]. Total lipids and carbohydrates from tissues were measured by colorimetric assays as previously described [21]. Briefly, the tissues were placed in either 500 μl of isopropanol (for lipid quantification) or 500 μl 10% cold trichloroacetic acid (TCA, for carbohydrate quantification), homogenized and then centrifuged for 10 min at 20°C and 8000 x g. For lipid quantification, 400 μl of the supernatants were transferred to 1.5 ml tubes containing 100 μL of 1 M KOH. Then, the tubes were incubated at 60°C for 10 min and once they were cool, 100 μl of sodium periodate solution (11.6 mM sodium periodate in 2 N glacial acetic acid) was added. After 10 min of incubation at room temperature, 600 μl of chromogenic solution (40 ml of 2 M ammonium acetate, 40 ml of isopropanol, 150 ml of acetyl acetone) were added to the samples and incubated for 30 min at 60°C. The resultant colour was measured at 410 nm using a plate reader spectrophotometer (Cytation 3 Imaging Reader, BioTek, Winooski, VT, USA). Using a commercial standard (T7531STD, by Pointe Scientific, Canton, MI, USA), a curve of triglycerides ranging from 0 to 60 μg was run independently and in parallel with the experimental samples. FB and OV carbohydrate content was measured using the anthrone colorimetric assay. Briefly, 50 μl of the supernatants after TCA precipitation were mixed with 500 μl of anthrone solution (26 mM anthrone, 1.31 mM thiourea, 66% sulfuric acid) and incubated for 20 min at 100°C. The samples were allowed to cool in the dark for 15 min and then quantified at 620 nm using a plate reader spectrophotometer described. A standard curve was run in parallel with the experimental samples using a 0–40 μg range of trehalose, which was dissolved in PBS. Protein quantification was done using the BCA protein quantification assay (Pierce BCA Protein Assay Kit by Thermo Fischer, ON, Canada). Three independent experiments were analyzed (n = 3) for each measurement with each n composed of tissues from 5 insects.

## Results and discussion

We were surprised to observe no major gene differences in the CNS between the UFC and FC; only 0.27% of the total number of genes detected in the CNS were regulated differentially between both nutritional states [17] but none of these were the focus of our currently analysis. Also, none of the GO functional terms were enriched in the CNS under these different nutritional states. We chose 3 days post-blood meal as the fed condition because of the morphological changes observed in the FB and OV [17]. The days chosen to monitor transcriptional regulation are appropriate for the FB and OV but apparently not for CNS. Neuropeptides play an important role in the regulation of reproduction and in insects are present in stereotypic patterns of neurons and neurosecretory cells in the CNS [22]. Using *R*. *prolixus* adults, Sterkel et al. [23] reported a quantitative proteomic analysis of the post-feeding response from CNS using 3 different conditions: unfed, 4 h and 24 h after blood intake. Only 4 neuropeptides (NVP-like, ITG-like, kinin-precursor peptide and NPLP1) were significantly regulated in response to the blood meal. Blood-feeding in *R*. *prolixus* leads to the release of neuropeptides involved in both short-term events such as rapid post-feeding diuresis, and long-term events, such as growth, molting or reproduction. For these latter events, neuropeptides are released and then re-stocked in neurosecretory cells and through their release into the hemolymph initiate the physiological changes observed in the FB and OV during the fed condition to promote egg growth. Therefore, when examining transcriptional regulation in the CNS it may be difficult to find a specific time point to detect differentially expressed transcripts in the CNS linked only to the vitellogenic process when comparing unfed and fed animals. For this reason, below, we focused our attention on the FB and OV and reflect on the CNS transcriptome analysis when making reference to peptide/hormone signaling.

To validate Illumina sequencing, 7 mRNAs were chosen and their relative transcript abundance in FB and OV in both nutritional states monitored by RT-qPCR. A good correlation was found between RNA-seq and RT-qPCR data; the Pearson tests were 0.9311 (to FB) and 0.9109 (to OV), with a statistical significance of *p* < 0.01 (S1 Fig). Multiples genes from these transcriptomes were also validated using RT-qPCR by Leyria et al. [17].

### GO enrichment analysis

**Fig 1:**
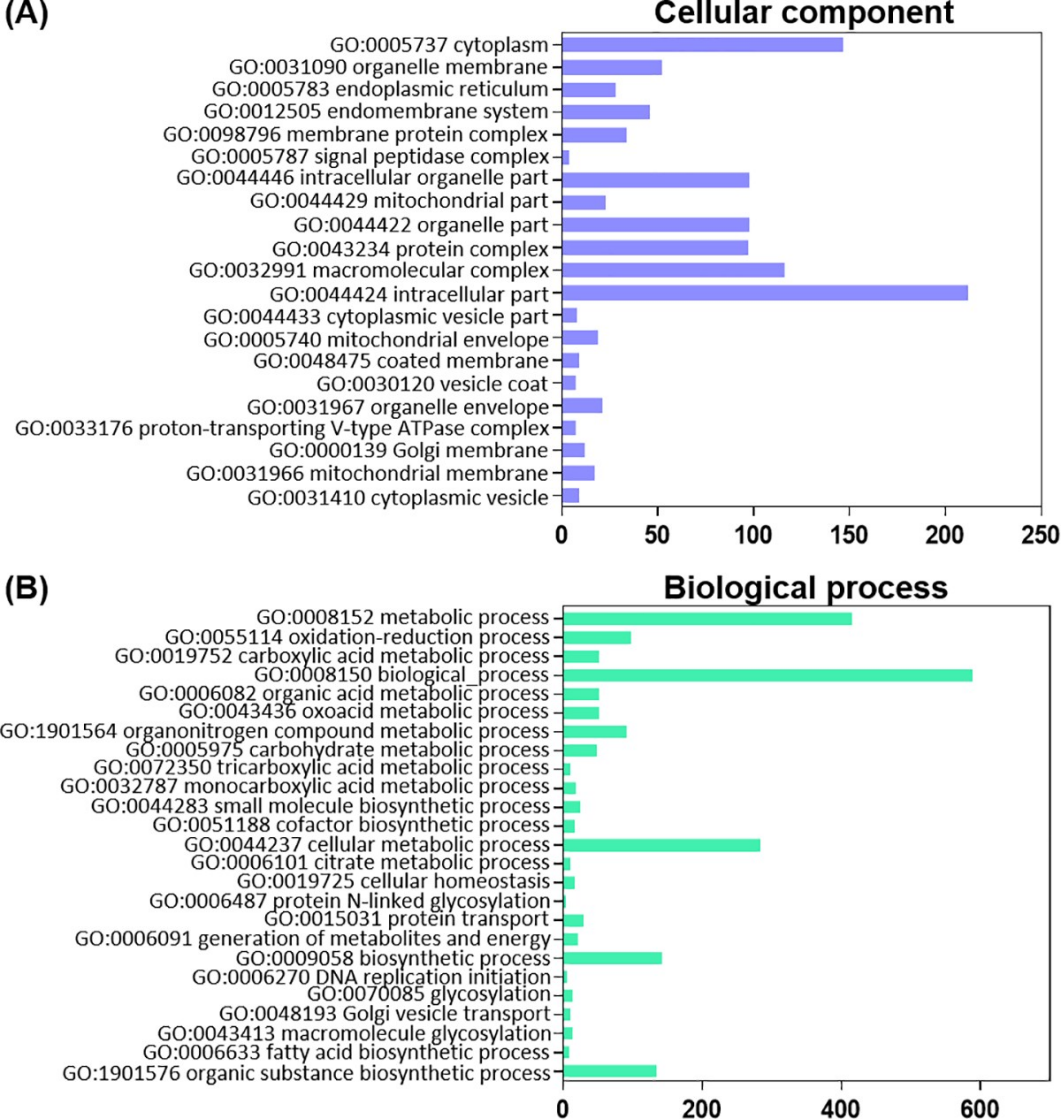
Distribution of differentially expressed genes (DEG) in the fat body annotated by GO enrichment analysis, associated with cellular components and biological processes. The GO enrichment bar chart presents the number of DEG enriched in cellular component (**A**) and biological process (**B**). The y-axis is GO terms enriched and the x-axis is the number of DEG. GO terms with corrected *P*-value less than 0.05 were considered significantly enriched in DEG (comparing FB_FC vs FB_UFC). The most significant enriched terms relevant to our analysis are shown. FB_FC, fat body in fed condition; FB_UFC, fat body in unfed condition.

**Fig 2:**
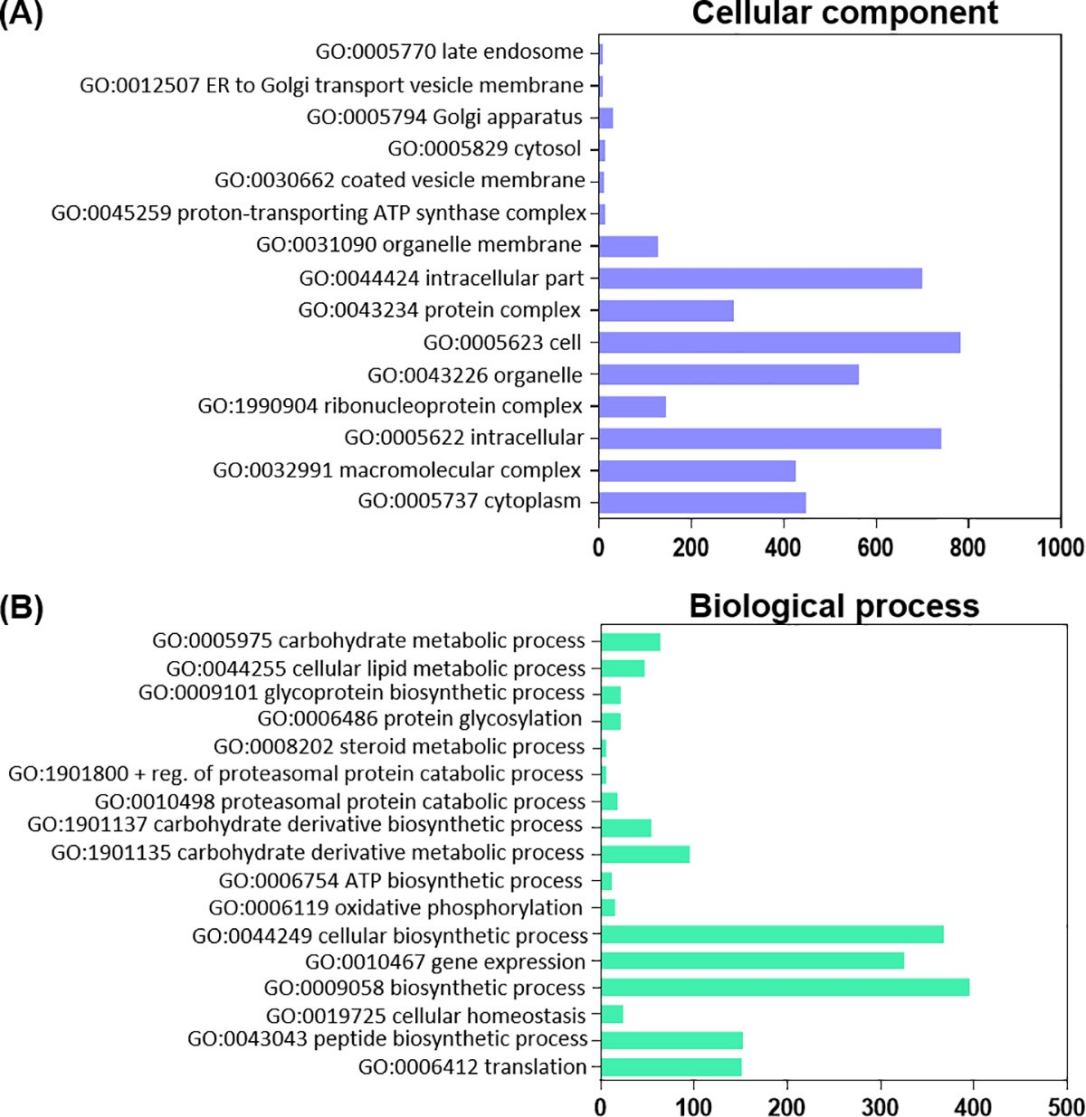
Distribution of differentially expressed genes (DEG) in ovaries annotated by GO enrichment analysis, associated with cellular components and biological processes. The GO enrichment bar chart presents the number of DEG enriched in cellular component (**A**) and biological process (**B**). The y-axis is GO terms enriched and the x-axis is the number of DEG. GO terms with corrected *P*-value less than 0.05 were considered significantly enriched in DEG (comparing OV_FC vs OV_UFC). The most significant enriched terms relevant to our analysis, are shown. OV_FC, ovary in fed condition; OV_UFC, ovary in unfed condition.

**Fig 3:**
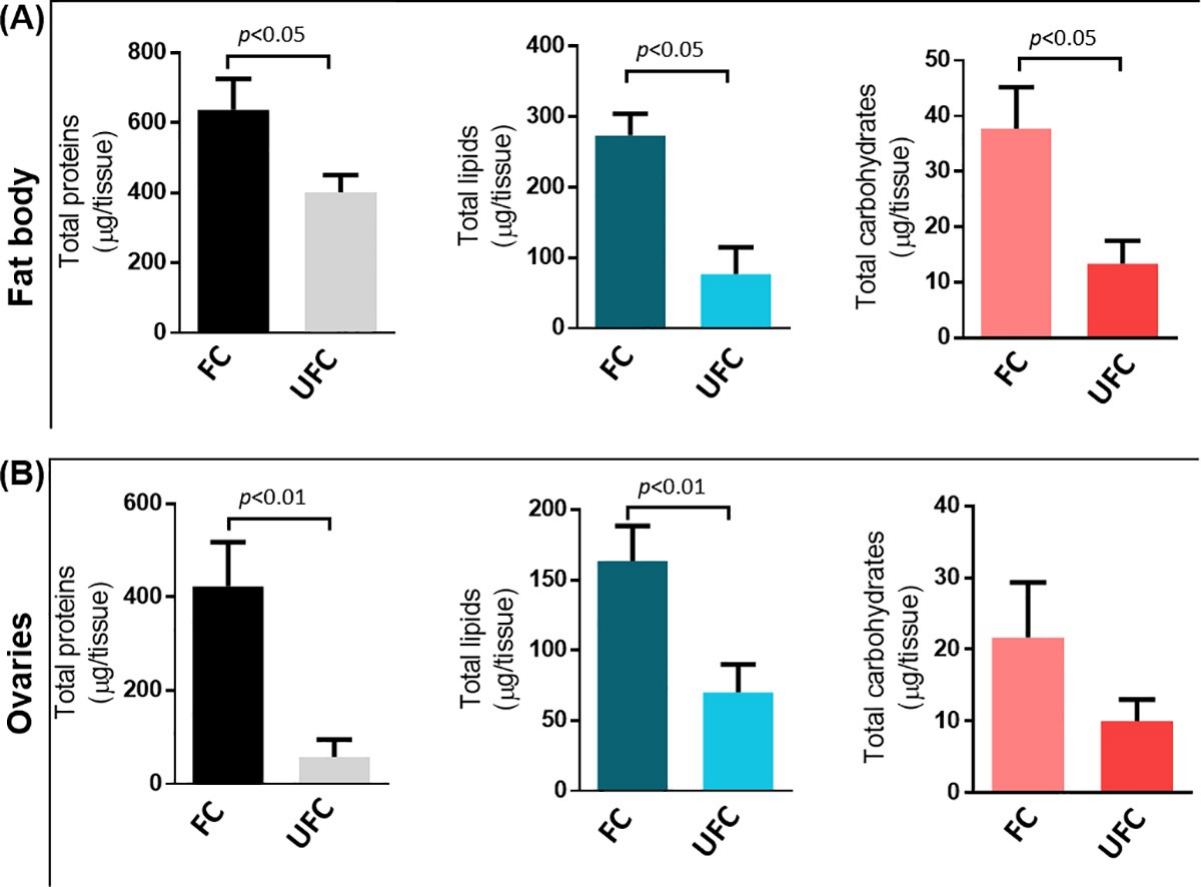
Protein, lipid and carbohydrate content in the fat body and ovaries of females during the unfed (UFC) and fed condition (FC). Fat body **(A)** and ovaries **(B)** were dissected from adult females during the unfed and fed condition. The tissues were homogenized and nutrient extracted and quantified as described in Materials and Methods. The results for total lipid, carbohydrate and protein content were graphed as the mean ± Standard Error of the Mean (SEM) from three independent experiments. Graphs and statistical tests were performed using GraphPad Prism 7 (GraphPad Software, CA, USA, www.graphpad.com). All datasets passed normality and homoscedasticity tests. The statistical significance of the data was calculated using Student's t-test. A P value < 0.05 was considered statistically significant.

Nutrients are essential for energy homeostasis of any organism and important changes in nutrient stores occur between feeding and non-feeding periods, more remarkably in adult insects during the reproductive process [9]. GO enrichment was used to assign a functional classification to DEGs. All DEGs categorize into two main groups: cellular components and biological processes. In cellular components, they are divided into 21 terms which are significantly up-regulated in FB_FC with respect to FB_UFC (Fig 1A). The most represented cellular component terms are cell parts involved in protein synthesis and secretory pathway, as it is to be expected since the FB is the main synthesis and secretory organ responsible for the production of virtually all hemolymph proteins. With regard to biological processes, terms involved in biosynthesis and lipid, carbohydrate and energy metabolism, are up-regulated in the FB_FC (Fig 1B). Recently, by examining KEGG enrichment we reported that the “ABC transporters pathway”, transporters which use energy to translocate substrates across cell membranes (e.g., sugar, lipid and peptides), is up-regulated in FB_FC, which shows that the synthesized nutrients are released during vitellogenesis mainly to be taken up by developing oocytes. In the OV, the main terms of cellular components and biological processes which are significantly up-regulated in OV_FC with respect to OV_UFC are related to lipid, carbohydrate and protein metabolism, and yolk granule formation (specialized structures which stores all nutrients used as substrates for embryogenesis and maintenance of the newly hatched nymph) (Fig 2A and 2B). These nutrients are mostly proteins, lipids and carbohydrates, produced by the FB, released into the hemolymph and subsequently taken up by the oocytes [24]. As we anticipated in light of the results of the GO enrichment, lipid, protein and carbohydrate levels in the FB and OV are increased in fed females (Fig 3A and 3B), as reported in *R*. *prolixus* by other authors and also reported in other vectors of Chagas’ disease [25–30]. In addition, it is clear that stored proteins are always the major component in both tissues, followed by lipids and then carbohydrate stores.

### Protein and hormone analysis

**Fig 4:**
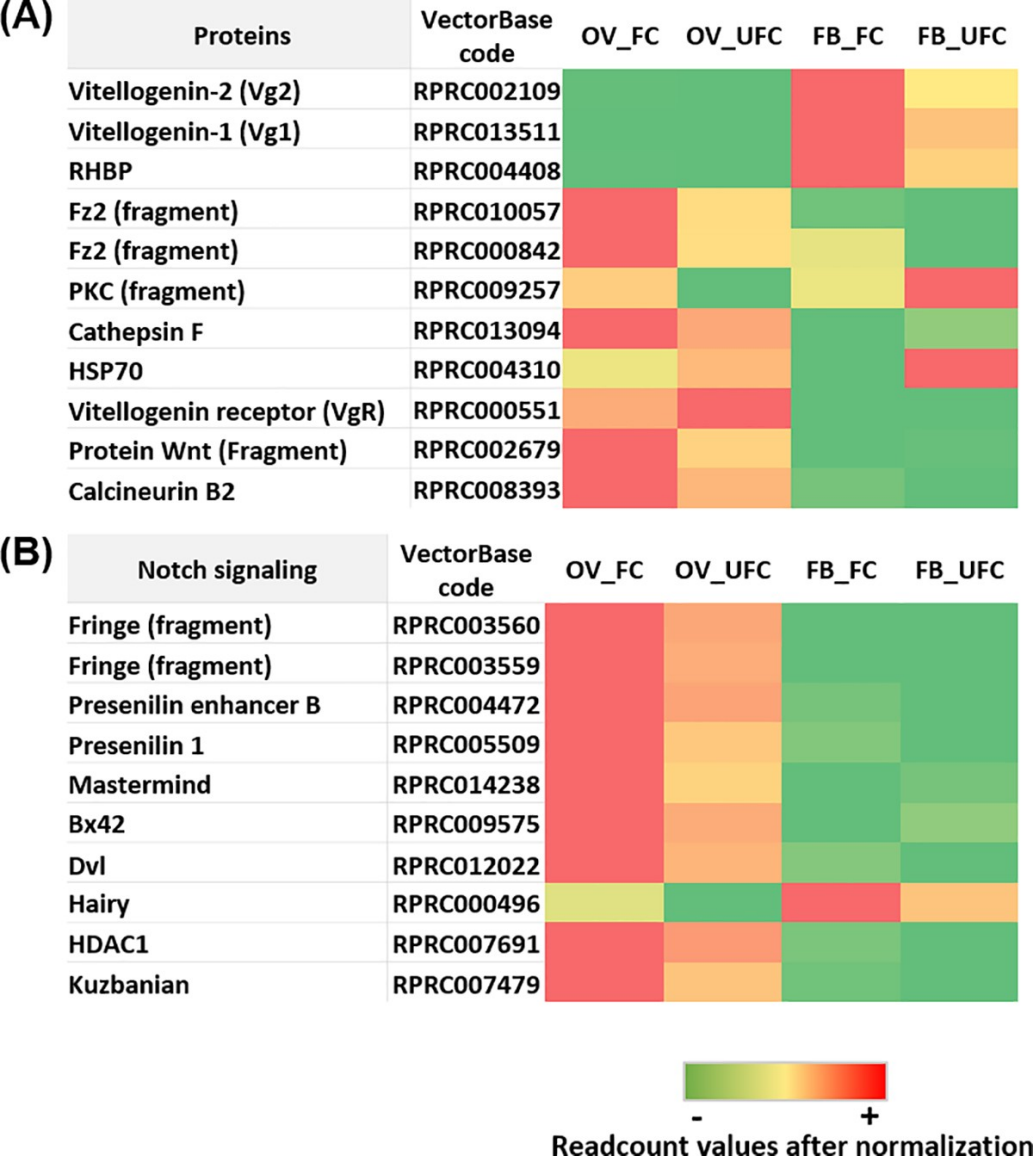
**Heat map comparing the mRNA expression levels of proteins related to reproduction (A) and Notch signaling pathway (B) in fat body and ovaries of females in different nutritional conditions.** The input data is the readcount value from the gene expression level analysis after normalization and is presented by means of a colour scale, in which green/yellow/red represent lowest/moderate/highest expression. DESeq was used to perform the analysis.

Vitellogenins (Vgs), the main yolk protein precursors (YPPs), are large molecules synthesized predominantly by the FB, secreted into the hemolymph and then transported to the OVs. The number of genes encoding insect Vgs varies from one to several depending on the species [31]. Our results show that mRNA levels for Vgs are considerable higher in the FB with respect to the OV, which is not surprising (Fig 4A). In the FB, transcripts levels for *Vg1* and *Vg2* increase after a blood meal, with *Vg1* having the highest expression; however, only *Vg2* mRNA is up-regulated at our time point which represents vitellogenesis (FB_FC) (Fig 4A and S1 Table). In *Triatoma infestans*, a triatomine related to *R*. *prolixus*, *Vg1* and *Vg2* genes are expressed at relatively low levels during the UFC and both Vg transcripts are up-regulated after blood-feeding [32]. During the reproductive phase, *T*. *infestans* shows patterns of expression of *Vg1* and *Vg2* with a bimodal distribution, displaying 2 expression peaks around both early and advanced vitellogenesis [32]. We cannot rule out the possibility that the levels of *Vg1* in the FB of *R*. *prolixus* also change as vitellogenesis progresses; however, we were unable to find a peak or up-regulation of *Vg1* at our chosen time point. In addition, even though there was no statistically significant difference, Vg1 expression in FB_FC is a fold higher than FB_UFC. Recently, by KEGG enrichment we reported “amino sugar and nucleotide sugar metabolism” and “N-Glycan biosynthesis” are pathways up-regulated in the FB of fed females [17]. Glycosylation is a critical post-translational modification to obtain the proper protein structure for adequate protein function and for Vgs, glycosylation is a step necessary for folding, processing and transport to the oocyte [33]. As previously reported in *R*. *prolixus* [34], our results suggest that Vg synthesis also could occur in the OV, with *Vg* transcripts up-regulated after a blood meal and *Vg1* levels higher than *Vg2* (Fig 4A and S1 Table). Interestingly, in *T*. *infestans* the *Vg2* transcript is quantitatively more important that *Vg1* in OVs of female insects after feeding [32]. As far as we are aware, there is no published phylogenetic analysis to see if *Vg1* and *Vg2* from *R*. *prolixus* are orthologues of *Vg1* and *Vg2* from *T*. *infestans*. Therefore, we performed a phylogenetic analysis and the results indicate that the same genes could have been given different names between the species (S2 Fig). The *vitellogenin receptor* (VgR) mRNA expression, the endocytic receptor responsible for Vg uptake by oocytes, is up-regulated in OV of unfed insects (Fig 4A and S1 Table), contrary to expectation since Vg uptake occurs after a blood meal. However, as expected the main KEGG enrichment pathways involved in receptor-mediated endocytosis signaling (endocytosis, lysosome and phagosome pathways) are enriched in OV_FC of *R*. *prolixus* [17]. This result indicates that even when the OV expresses high endocytic receptor transcript levels in the UFC, only after a blood meal does the endocytic process occur. In female triatomines, the ovarioles exhibit typical asynchronous development [24] and can produce 30 to 45 eggs over 20–30 days following each blood meal. Therefore, during the vitellogenic period there are oocytes in varying stages of development, and VgR expression will be required throughout that period. We suggest an up-regulation of *VgR* transcript expression could be necessary to store mRNA during the pre-vitellogenic state to support a translation regulation of VgR during vitellogenesis. In the cockroaches, *Periplaneta americana* and *Blattella germanica*, a VgR translation control was reported during vitellogenesis [35, 36]. Also, a recycling of the VgR protein might be occurring, as reported in mosquitoes [37], and therefore an increase in transcriptional regulation would not be necessary during vitellogenesis and the VgR protein expression by OVs to allow uptake of Vg is supported by recycling. A similar pattern of high VgR mRNA levels in non-reproductive stages and low levels during vitellogenesis is found not only in insects but also in oviparous vertebrates [38, 39]. On the other hand, using *R*. *prolixus* females, Oliveira et al. [40] described another YPP, a 15-kDa protein called *Rhodnius* heme binding protein (RHBP), which works as an antioxidant agent in hemolymph. After the blood meal, a large amount of heme is released from hemoglobin, crosses the digestive barrier and reaches the hemolymph, where it is sequestered by RHBP. Here, we show that in the FB, *RHBP* mRNA levels are up-regulated in females 3 days after feeding (Fig 4A and S1 Table). The increase of synthesis of YPPs in FB_FC coincides with the KEGG analysis reported recently, where we show an enrichment of “biosynthesis of amino acids” and “protein processing in endoplasmic reticulum”, a pathway that includes four major protein processing roles: folding/refolding of the polypeptide, glycosylation of the protein, assembly of multi-subunit proteins, and packaging of proteins into vesicles [17]. Even knowing that blood is a rich source of amino acids for yolk protein precursor synthesis, we cannot rule out that *de novo* synthesis of amino acids by FB_FC is relevant during the vitellogenic process (S4 Table).

As it is widely known, vertebrate blood is richer in protein than lipid and carbohydrate, and so blood-feeding insects must use amino acids derived from the blood meal to produce lipid and carbohydrate. These processes result in the release of nitrogen as ammonia. In mosquitoes, when the rate of ammonia production exceeds the capacity to produce urea and/or uric acid, the additional ammonia is stored temporarily as proline. Eventually, ammonia is recovered from proline and excreted, and the carbon skeleton is used for lipid and carbohydrate synthesis or as a source for energy production [41]. In this context, several studies indicate that proline can be used as an energy substrate, mostly by flight muscle, in several insect species [42, 43]. However, as far as we know, there are no reports in the literature indicating that proline might play a role during vitellogenesis. As *R*. *prolixus* is also a blood-sucking insect, several mechanisms of blood processing could be common with female mosquitoes. Interesting, by KEGG we find arginine and proline metabolism are up-regulated in FB after a blood meal (S4 Table). In mosquitoes, the increase in proline levels in the hemolymph after blood ingestion was not only derived from dietary proline, but also synthetized *de novo* [41]. Here, the up-regulation of amino acids biosynthesis found in FB_FC of *R*. *prolixus* females could, in part, be responsible for proline synthesis. In the S1 Table, we show that two enzymes involved in proline synthesis, pyrroline-5-carboxylate reductase-like (P5CR) and delta-1-pyrroline-5-carboxylate synthase-like (P5CS) are increased in the FB after a blood meal. In addition, cytosol aminopeptidase like (LAP3R) and proline dehydrogenase like (ProDH), both involved with proline degradation, are also up-regulated or increased in FB_FC (S1 Table). Overall, these results indicate that proline could be synthesized and degraded to contribute to the energy requirement of the FB during the vitellogenic process and/or to be used as a nitrogen sink during blood meal digestion, as reported in mosquitoes [41]. We also show that glutamate synthase, an enzyme which catalyses glutamine conversion to glutamate, and P5CS are up-regulated in OV_FC. Glutamate can be used for proline synthesis and/or as an energy source via tricarboxylic acid cycle. Therefore, proline metabolism also could contributed to energy required by OV_FC during vitellogenesis.

**Fig 5:**
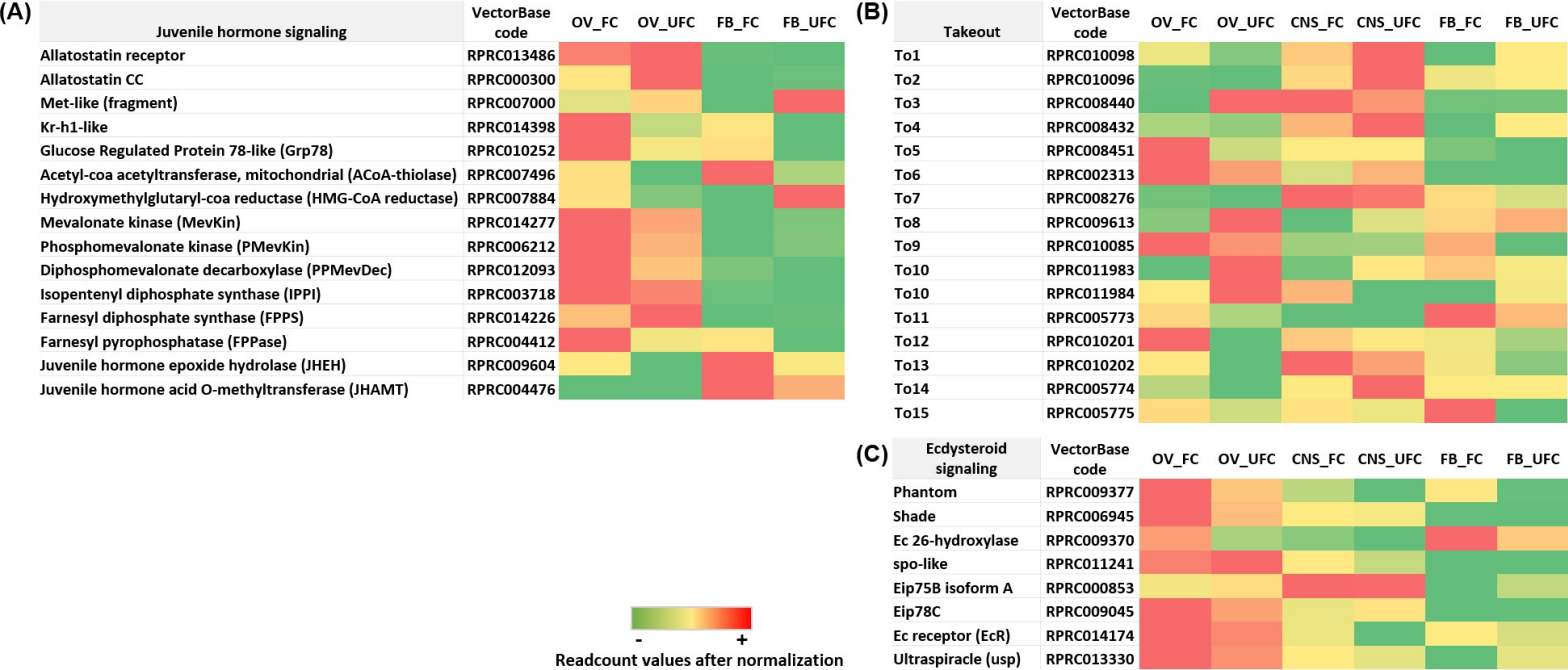
**Heat map comparing the mRNA expression levels of molecules involved in juvenile hormone signaling (A), *takeout* genes (B), and ecdysteroid signaling (C) in fat body and ovaries of female adults in different nutritional condition.** The input data is the readcount value from the gene expression level analysis after normalization and is presented by means of a colour scale, in which green/yellow/red represent lowest/moderate/highest expression. DESeq was used to perform the analysis.

The Wnt signaling pathway was first discovered as a key event in *D*. *melanogaster* development [44]. The Wnt (glycoprotein ligand) and Frizzled (Fz, transmembrane Wnt receptor) proteins interact with structural components at the cell surface to initiate the signaling cascades that result in transcriptional regulation of gene expression. In *A*. *aegypti*, a fundamental role of Fz2 was reported in egg production [45]. Here, we find that *Wnt* and *Fz2* mRNA levels are up-regulated in OV_FC (Fig 4A and S1 Table). Additionally, Wnt and ToR signaling interact synergistically in the vitellogenic process [45] and supporting this finding, we showed ToR signaling is active in OV_FC [17]. Also, the non-canonical Wnt pathway indicates that Wnt/Fz signaling leads to the release of intracellular calcium through trimeric G proteins [45]. The calcium release and intracellular accumulation activates several Ca^2+^- sensitive proteins, including protein kinase C (PKC), calcineurin and calcium/calmodulin-dependent kinase II (CamKII). In *A*. *aegypti* it was found that juvenile hormone (JH) activates the phospholipase C (PLC) pathway and quickly increases the levels of Ca^2+^ for the activation and autophosphorylation of CaMKII, which is involved in patency development [46]. On the other hand, it was reported that an increase in intracellular Ca^2+^ levels induce egg activation, the process by which an oocyte is prepared for embryogenesis [47]. In this sense, by genetic studies were reported essential roles for the calcium-dependent enzyme calcineurin in *Drosophila* egg activation [48]. By DEG analysis, we demonstrate an up-regulation of *PKC* and *calcineurin* in OV from fed insects (Fig 4A and S1 Table). In *R*. *prolixus*, earlier studies by Ilenchuk et al. [49] suggested that a PKC might be involved in patency and Vg uptake but until now the receptors or molecular mechanisms responsible for this cascade are unknown. The results we observe in vitellogenic oocytes of *R*. *prolixus* could be indicative of a relationship between patency and Wnt/Fz2/Ca^2+^ signaling. Methoprene-tolerant (Met), which encodes a transcription factor of the bHLH-PAS family, was reported to be a JH receptor [50]. *Krüppel homolog 1* (Kr-h1), identified as the main JH primary-response gene activated by Met [50], is up-regulated in OV_FC (Fig 5A and S1 Table), which supports the hypothesis that in *R*. *prolixus*, JH is working directly on OVs possibly to stimulate egg formation.

Heat shock proteins represent different protein families based on their sequence homology and molecular masses. Among them, Heat shock protein 70 family (Hsp70) is highly conserved between species [51]. The expression of Hsp70 is considered a good marker for the inducible stress response in an organism [52]. In *T*. *infestan*s Hsp70 is strongly expressed in unfed insects [53]. Similarly, in *R*. *prolixus*, we find that *Hsp70* is up-regulated in the FB from unfed females (Fig 4A and S1 Table), a condition inherently associated with a stressful situation. Glucose-regulated protein of 78 kDa (Grp78) is a member of the Hsp70 family which acts as a chaperone to facilitate protein folding and to inhibit protein aggregation of new peptides. Interestingly, in *Locusta migratoria*, Grp78 was reported as a regulatory factor of Vg synthesis and cell homeostasis in the FB via JH signaling [54]. In *R*. *prolixus*, we show a significant up-regulation of Grp78-like protein in both FB and OV of fed insects (Fig 5A and S1 Table). This result suggests a novel regulatory mechanism involved in the vitellogenic process of *R*. *prolixus*.

Notch is a receptor that directly translates information of cell-cell contact to gene expression in the nucleus [55]. In *D*. *melanogaster* adult female, Notch is required to the differentiation of all epithelial follicle cells and the establishment of anterior-posterior polarity in the oocyte [56]. Also, in *B*. *germanica*, it was demonstrated that Notch is important in maintaining the proliferative and non-apoptotic state of follicular cells, as well as, in differentiation of the posterior follicular cell population [57]. By KEGG analysis, we showed that *Notch signaling* is up-regulated in the OV of fed females [17]. Here, we find that transcripts involved in Notch developmental functions, such as Fridge, presenilin enhancer 2 (PEN-2) and presenilin*-*1, are up-regulated in OV_FC (Fig 4B and S1 Table). Mastermind is an essential nuclear factor that supports the activity of Notch [58]. In OV_FC of *R*. *prolixus*, *mastermind* transcriptional factor is also up-regulated, as well as Bx42 (Fig 4B and S1 Table), an essential factor which, via Notch, is involved in the formation of different tissues during embryogenesis [59]. *Kuzbanian*, a key regulator of the Notch signaling pathway, is essential for border cell migration in the *D*. *melanogaster* ovary [60]. In *R*. *prolixus*, we find *Kuzbanian* mRNA up-regulated in OV_FC, as well as the histone deacetylase HDAC1, a positive regulator of Notch signaling [61]. In addition, we find up-regulation of *Hairy* gene, the most extensively studied and best understood target of Notch signaling [62]. Overall, it is likely that the up-regulation of Notch signaling in OV *R*. *prolixus* after a blood meal is related mainly with follicular cell metabolism during egg growth.

During vitellogenesis, JH titres are expected to increase, since this is one of the main hormones involved in Vg synthesis. Eight different forms of JH have been identified so far, with JH III the most widely distributed among insects [50]. Recently, the JH in *R*. *prolixus* was found to be JH III skipped bisepoxide (JHSB3) [63]. Although all JH homologs have structural differences, they share a common biosynthetic pathway which involve basically 13 enzymatic reactions and is conventionally divided into early (the mevalonate pathway, MVAP) and late (JH-branch) steps [50]. Although the known site of *de novo* JH biosynthesis is the *corpora allata* (CA), we cannot rule out that other tissues are capable of synthesizing JH. By KEGG analysis, two pathways related to JH, “Insect hormone biosynthesis” and “Terpenoid backbone biosynthesis”, are up-regulated in the FB and OV during the FC [17]. Here, we find 5 enzymes involved in the MVAP are up-regulated in the OV after a blood meal; acetyl-coa acetyltransferase, mitochondrial (ACoA-thiolase), hydroxymethylglutaryl-coa reductase (HMG-CoA reductase), mevalonate kinase (MevKin), phosphomevalonate kinase (P-Mevkin) and diphosphomevalonate decarboxylase (PP-MevDec). In FB, only (ACoA-thiolase) is up-regulated after feeding. In Diptera, Hymenoptera, Blattodea and Lepidoptera, genes involved with the MVAP are expressed not only in the CA but also in other tissues such as the OV and FB [64–67]. It is interesting to note that the enzymes involved in the early steps of the MVAP are also responsible for the production of other terpenoids such as defensive secretions and pheromones [68, 69]. Farnesyl pyrophosphatase (FPPase), JH acid methyltransferase (JHAMT) and JH epoxide hydrolase (EPOX) are enzymes that convert JH acid or inactive precursors of JH to active JHs at the late step of the JH biosynthesis pathway in insects [50]. Here, we find that all of these are present in the FB and OV with FPPase and EPOX up-regulated in OV_FC whereas just FPPase is up-regulated in FB_FC (Fig 5A and S1 Table). A small amount of JHAMT was also found in the OV and FB of *Bombyx mori* and *Helicoverpa armigera*, respectively [70]. In addition, JH I was originally isolated from the abdomens of male pupae of *Hyalophora cecropia* [71]. Also, in male *Cecropia* moth, JH was reported to be synthesised by the male accessory glands from JH acid secreted by the CA [72]. In mosquitoes the male accessory glands and OV are able to synthesize JH [73, 74] and, indeed, it has been shown that male accessory glands transfer JH to females at mating [75]. Recently, it was shown that JHs can also be synthesized by the adult *D*. *melanogaster* gut [76]. In this context, it is important to highlight that *R*. *prolixus* allatectomized immediately after emergence as adults, continue to make a few eggs [77]. This finding also may indicate an alternate source of JH. Overall, our results suggest that most vitellogenic OV but also FB, may have the potential of synthesizing JH in *R*. *prolixus*.

In addition, insect cytochrome P450s include a group of different enzymes involved in detoxication and biosynthesis of ecdysteroids and JH [78, 79]. Previously, by KEGG analysis, we reported an up-regulation of metabolism of xenobiotics by cytochrome P450 in FB_FC, possibly because of an increase in hormone synthesis or/and a detoxification after a blood meal [17]. Allatostatin-C (ASTC) is a family of peptides originally associated with the control of CA activity but now known to be pleiotropic. ASTC and its paralog, ASTCC, are very similar peptides, likely generated by gene duplication, and their receptors possibly have a common ancestor as well [80]. We find a significant up-regulation of *ASTCC* mRNA expression in OV_UFC (Fig 5A and S1 Table), but so far, there is no evidence about the specific role of this peptide on OVs.

JH is transported from the site of synthesis to target tissues by a hemolymph carrier protein called juvenile hormone-binding protein (JHBP). JHBP protects JH molecules from hydrolysis by esterases present in the insect hemolymph [81]. The takeout genes (*To*) were discovered as a circadian-regulated gene and belong to the JHBPs family [82]. The *To* genes modulate various physiological processes, such as behavioral plasticity in the migratory locust *L*. *migratoria* and feeding in *D*. *melanogaster* [83, 84]. In the brown planthopper *Nilaparvata lugens*, the *To* family of genes were reported to be regulated by JH signaling [85]. Fifteen such genes were identified in the antenna of *R*. *prolixus* [86]. Here, we find that *To* genes have a unique pattern of expression according to the tissue analyzed and feeding condition (Fig 5B and S1 Table). *To1*, *To2*, *To4* and *To7* mRNA expression is highly expressed in the CNS of unfed insects, suggesting that starvation could induce the expression of these genes. In addition, while *To9*, *To11*, *To12* and *To15* mRNA expression is significantly increased in the FB from females after a blood meal, *To5*, *To12* and *To13* transcripts show a significantly increased expression in OV_FC (Fig 5B and S1 Table). This is the first report of an analysis of *To* genes in different tissues involved in reproduction in *R*. *prolixus*, providing new insights into the mechanisms involved in egg formation.

Ecdysteroids are also critical developmental hormones involved in the regulation of molting and metamorphosis. The prothoracic glands (PGs) are the major source of these ecdysteroids in larvae, but PGs usually degenerate prior to the early adult stage, where alternative sites of ecdysteroid production have been described [87]. Cardinal-Aucoin et al. [88] reported that in *R*. *prolixus*, between days 3 and 4 after a blood meal, ovarian ecdysteroid content increased 4–5 fold to a level that was sustained for the duration of egg development. This pattern is similar to that seen in the hemolymph ecdysteroid titer. Two interpretations were proposed a) the ovary passively absorbs hemolymph ecdysteroids or b) the ovary produces the ecdysteroids found in the hemolymph. After a blood meal, we find up-regulation of 3 enzymes involved in ecdysteroid synthesis in the OV, *Shade*, *Phantom* and *26-hydroxilase*, supporting the second hypothesis (Fig 5C and S1 Table). Garcia et al. [89] showed that inhibition of JH biosynthesis in *R*. *prolixus* females induces an increase in ecdysteroid synthesis by the OV. Also, Ruegg et al. [90] reported that hemolymph titres of ecdysteroids in *R*. *prolixus* females peak 5 days after a blood meal and that ovariectomy prevents that increase, demonstrating an influence by the OV on ecdysteroid production. Coincidently, ecdysteroid biosynthesis by the OV in *D*. *melanogaster*, *A*. *aegypti* and *Nilaparvata lugens* has already been reported [91–94]. Ecdysteroid signaling involves the activation of a heterodimer receptor, composed of the ecdysone receptor (EcR) and the ultraspiracle protein (USP) [95]. Here, we find up-regulation of USP and no statistically significant increase in the EcR in the OVs after a blood meal. In *Tribolium castaneum*, an insect with the same type of ovaries (telotrophic meroistic) as *R*. *prolixus*, 20-hydroxyecdysone (20E) and its receptors are required for ovarian growth, oocyte maturation and follicle cell differentiation [96]. Overall, we suggest that OVs of *R*. *prolixus* females are not only a source for ecdysteroid synthesis but also that activation of ecdysteroid signaling could be key in ovarian development.

### Carbohydrate analysis

**Fig 6:**
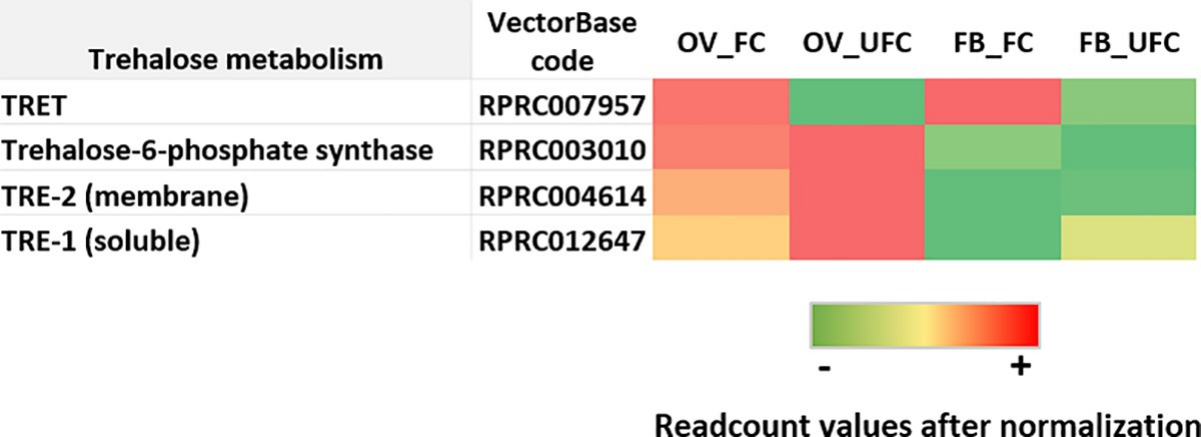
Heat map comparing the mRNA expression levels of molecules involved in trehalose metabolism in fat body and ovaries of female adults in different nutritional condition. The input data is the readcount value from the gene expression level analysis after normalization and is presented by means of a colour scale, in which green/yellow/red represent lowest/moderate/highest expression. DESeq was used to perform the analysis.

The main blood sugar in insects is trehalose, a sugar that consists of two glycosidically linked glucose units. Trehalose homeostasis is controlled by trehalose-6-phosphate synthase, the main enzyme involved in trehalose synthesis by the FB; trehalose transporter (TRET), which has a particular direction of transport depending on the trehalose gradient, and trehalases, specifically two isoforms, soluble (TRE-1) and membrane-bound (TRE-2), involved in the conversion of trehalose to glucose to generate energy [97, 98]. DEG analysis reveals that *trehalose-6-phosphate synthase* and *TRET* are up-regulated in the FB during the FC (Fig 6 and S1 Table). It is widely accepted that the vitellogenic process is an event with high energy demands. Thus, trehalose synthesis and release after a blood meal from FB to circulation, could be necessary steps to trehalose uptake by developing oocytes, which accumulate carbohydrates as a resource for embryogenesis [24]. Indeed, Santos et al. [25] revealed the importance of carbohydrate accumulation by oocytes for reproductive success. In a fertilized egg of *R*. *prolixus*, 70% of its glycogen is consumed mostly during early embryogenesis [25]. Supporting this finding, specific *phospholipase A2-like* mRNA (RPRC008617) is up-regulated in FB_FC (S1 Table). This belongs to a group of enzymes that are involved in either the formation or/and release of trehalose from FB cells [99]. In addition, we find that *trehalose-6-phosphate synthase* shows no change in the OVs when both nutritional conditions are compared (Fig 6 and S1 Table). Therefore, the trehalose that is uptake by OVs to induce glycogen synthesis must be incorporated from extra-ovarian sources. In *R*. *prolixus*, it has been suggested that in the OV, TRE-2 could interact with trehalose in the hemolymph supporting the idea that hydrolysis of trehalose at the cellular surface could be an obligatory step to provide glucose for carbohydrate accumulation by oocytes [100]. The researchers found that trehalase activity seemed not to be regulated at the transcriptional level after a blood meal. In addition, here we find that *TRE-2* is up-regulated in OVs but in unfed females (Fig 6 and S1 Table). We hypothesize that glucose obtained by the breakdown of trehalose could participate in the regulation of the energy necessary (contributed by different tissues, including OVs) to maintain overall metabolism of the insect until physiological conditions improve, as have been suggested to another triatomine [29]. An interesting finding from our results is that *TRET* is more than 6-fold up-regulated in OVs of fed insects (Fig 6 and S1 Table), supporting the hypothesis that a direct trehalose uptake from the hemolymph via TRET could be an important process involved in the storage of carbohydrates in ovaries.

### Lipid analysis

In insects the majority of lipid reserves are found in the FB as triacylglycerol (TAG). Lipids are critical to support situations of high metabolic demand, such as vitellogenesis [9]. In FB, TAG storage is mainly the result of 2 mechanisms: a) the transfer of dietary fat from the midgut to the FB by lipophorin (Lp), the main lipoprotein of insects, during feeding; and b) the synthesis of lipids from other nutrient reserves, such as amino acids or carbohydrates. In times of energy need, such as egg development, TAG reserves in the FB are mobilized and transported to target organs mainly in the form of DAG via Lp [9, 10]. Lipids in oocytes are important not only to provide energy but also for metabolic processes, plasticity of cell boundaries, intracellular signaling, and cuticle formation, among others. [23]. Energetically speaking, after oviposition about 40% of TAG and 72% of glycogen reserves are used during embryogenesis in *R*. *prolixus* [25, 27]. However, the complete oxidation of carbohydrates yields about 4 kcal/g, whereas the oxidation of fatty acids (FAs) yields about 9 kcal/g [9], further indicating the reason lipids are used as a major energy reservoir. Also, we cannot rule out that glycolysis or proline metabolism in OV_FC could contribute to energy production during vitellogenesis. Not only is glycolysis a pathway up-regulated in OV_FC (S5 Table) [17], but also LAP3R, ProDH and P5CDH, enzymes involved in proline catabolism, are up-regulated or increased in the OV during the fed condition (S1 Table). Therefore, even knowing that lipid represents a major source of energy in insects, we cannot ignore the contribution by glycolysis or proline metabolism.

**Fig 7:**
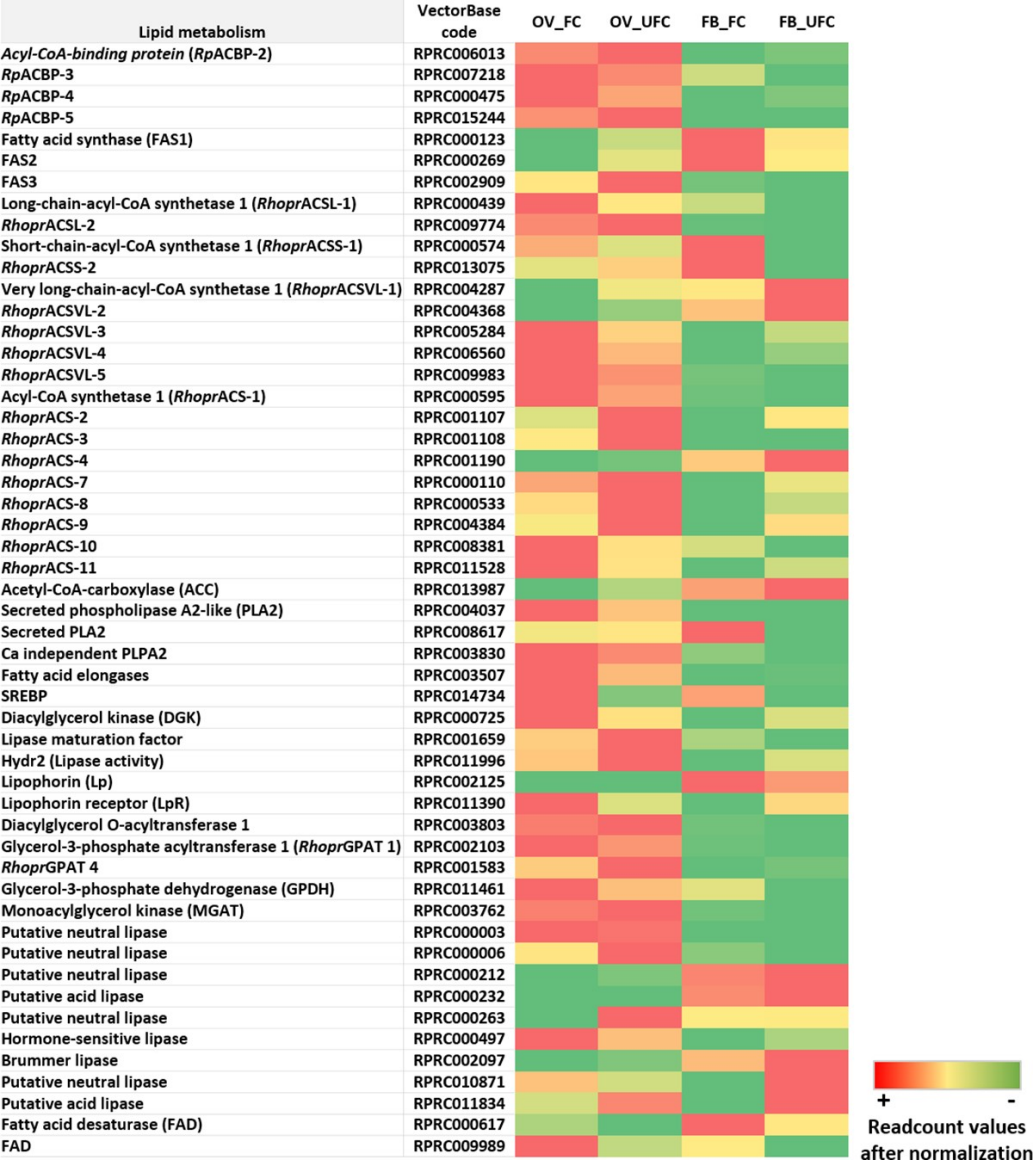
Heat map comparing the mRNA expression levels of molecules involved in lipid metabolism in fat body and ovaries of female adults in different nutritional condition. The input data is the readcount value from the gene expression level analysis after normalization and is presented by means of a colour scale, in which green/yellow/red represent lowest/moderate/highest expression. DESeq was used to do the analysis.

As the ability of insect oocytes to obtain fatty acids by *de novo* synthesis is very small, most of the lipids in the oocyte come from the FB via the hemolymph using Lp as transport [101]. In vitellogenesis, lipid accumulation by OVs is associated with a considerable reduction in the lipid content of the FB [9]. However, after a large blood meal, the triatomines must store a vast amount of TAG to support a possible period of fasting. This reality promotes a fine balance between lipid mobilization for egg growth and lipid storage to survive starvation. Here, we demonstrate that there are different types and subtypes of enzymes involved in lipid metabolism, as reported by Gondim et al. [102], and each one seems to have a particular role according to the specific tissue and physiological condition. TAG can be synthesized essentially by 2 different pathways, the monoacylglycerol (MG)-pathway and the glycerol-3 phosphate (G3P) pathway [9]. In *R*. *prolixus*, only the G3P pathway has been reported [103]. This pathway starts with acylation of G3P, catalyzed by G3P acyl transferases (GPAT). Two GPAT, *Rhopr*GPAT1 and *Rhopr*GPAT4, have already been described and characterized in *R*. *prolixus* [30, 103]. *Rhopr*GPAT1 expression is higher in the FB whereas *Rhopr*GPAT4 shows no change between OV and FB [30]. Also, it has been shown that *RhoprGPAT1* mRNA expression is up-regulated after a blood meal in the FB whereas *Rhopr*GPAT4 shows no changes throughout the different time points analyzed [30, 103]. However, we find that the mRNA expression of *RhoprGPA1* and *RhoprGPA4* is predominantly increased in the OVs with respect to the FB and only *RhoprGPAT4* is up-regulated in the OVs of unfed insects (Fig 7 and S1 Table). Nevertheless, Alvez-Bezerra et al. [30] suggested post-transcriptional mechanisms which could be involved in *Rhopr*GPAT activity. Taken together, the differences observed could be attributed to different conditions of insect rearing and feeding as well as the time points of the experimental insects, e.g. our experimental insects were females during the first reproductive cycle (after the first blood meal as an adult insect) or females at 10 days post-ecdysis (without a blood meal as an adult female) whereas Alves-Bezerra and Gondim [103] and Alves-Bezerra et al. [30] used adult females after the second or third meal, or starving females 3 weeks after the first or second blood meal. In addition, transcripts for enzymes involved with the synthesis and elongation of lipids, such as *insect microsomal and cytosolic fatty acid synthases (FAS1 and FAS2)*, *lipid elongases and sterol regulatory element-binding protein (SREBP)* are up-regulated in the FB after a blood meal (Fig 7 and S1 Table). These finding coincide with our previous report, where we show that both, “fatty acid biosynthesis” and “fatty acid elongation”, are KEGG pathways enriched in FB_FC [17]. Fatty acid desaturases (FAD) are essentials for *de novo* FA synthesis. In *R*. *prolixus* we show that 2 transcripts encoding for *FAD* are up-regulated in both FB and OV of fed insects (Fig 7 and S1 Table). These results suggest that after a blood meal, FA synthesis increases and confirms that, besides incorporation of lipids from hemolymph, *de novo* synthesis of FAs by the FB of *R*. *prolixus* occurs, as was suggested by Pontes et al. [26]. Therefore, FAs could be used to synthesize TAG, phospholipids or be oxidized for ATP production. For any of these pathways, FAs need to be activated which is the role of acyl CoA synthetases (ACS). In *R*. *prolixus*, 20 putative genes coding for ACS proteins have been reported [104]. Here, we report the mRNA expression of different *ACS* transcripts that encode short-chain ACS (ACSS), regular ACS, long-chain ACS (ACSL) and very long chain ACS (ACSVL). All these enzymes are present in both the FB and OV, but their expression patterns depend on the nutritional condition (Fig 7 and S1 Table). *Rhopr*ACS3, *Rhopr*ACS8 and *Rhopr*ACS9 are up-regulated in OV_UFC, and *Rhopr*ACSVL1, *Rhopr*ACSVL3, *Rhopr*ACS7, *Rhopr*ACS8, *Rhopr*ACS9, *Rhopr*ACS11 in FB_UFC, suggesting that β-oxidation is a pathway which in unfed *R*. *prolixus* females, could promote the synthesis of ATP as an energy source (Fig 7 and S1 Table). However, we cannot ignore the potential participation of ACS in lipid synthesis during the FC; *Rhopr*ACSL1, *Rhopr*ACSVL3 and *Rhopr*ACS11 are up-regulated in OV_FC and only *Rhopr*ACSS2 in FB_FC. For FA mobilization, lipases play a critical role to catalyze the hydrolysis of TAG molecules [9]. In this sense, transcripts related to lipid breakdown (*lipases*) or lipid transfer (*lipophorin receptor*, *LpR*) in general are increased in the FB of unfed insects (Fig 7 and S1 Table). Among others, we also find an increase (not statistically significant) of mRNA expression of *Hormone-sensitive lipase-like* and *Brummer lipase-like*, a homolog of human adipocyte triglyceride lipase, in the FB_UFC. Hormone-sensitive lipase is present in the lipid droplet of *D*. *melanogaster* and is involved in FB lipid mobilization during starvation [105]. Interestingly, in *D*. *melanogaster*, Brummer lipase is induced in the FB during starvation by FoxO-signaling [106], playing an important role in the metabolism of energy. Recently we reported that FoxO signaling is also up-regulated in FB_UFC of *R*. *prolixus* [17]. However, in *N*. *lugens*, a deficiency of *Brummer lipase* during vitellogenesis impairs lipid mobilization, negatively affecting egg production [107]. The reality that Brummer lipase mRNA expression show only a small increase during UFC respect to FC (statistically no significant, S1 Table), could be due to the fact that in *R*. *prolixus* this enzyme is necessary in both nutritional conditions, showing its pleiotropic effect. In addition, the lipase maturation factor 1 is a protein involved in the post-translational maturation of secreted homodimeric lipases [108]. In times of high energy demand, such as starvation, insects use TAG stores via the coordinated action of lipases. In our experiment, *lipase maturation factor* transcript expression is up-regulated in OV_UFC, as is the expression of *Hydr2* (*lipase activity enzyme)*, among other lipases (Fig 7 and S1 Table). These findings are another indication of the fine cross-talk between lipid synthesis and mobilization in both nutritional conditions.

Given the premise that oocytes have a low capacity to synthesize lipids *de novo*, it is surprising to find that *FAS2*, *FAS3* and *Acetyl CoA carboxylase (ACC)* mRNAs, which are lipogenic enzymes involved in *de novo* synthesis of FA, are up-regulated in OV_UFC (Fig 7 and S1 Table). Recently, we reported via KEGG analysis an up-regulation of “fatty acid biosynthesis pathway” in OV_UFC [17]. In mosquitoes, a FAS is also more highly expressed in diapause-destined females than in non-diapausing individuals [109]. Massive endocytosis of YPPs in oocyte and intense VgR, LpR and heavy-chain clathrin synthesis are all energy-dependent processes [110]; for that reason, lipid reserves in pre-vitellogenic oocytes (UFC) could also play a critical role in supporting the energetic demands of the growing oocyte at the beginning of vitellogenesis. On the other hand, in *A*. *aegypti*, deficiencies in ACC and FAS result in defects in eggshell formation [111]. The insect eggshell is a multilayered structure, secreted by the follicular cells, that confers physical and biological protection to the embryo during development [112]. Thus, the expression of *ACC* and *FAS* in the OV of blood-feeding *R*. *prolixus* females suggests an *in-situ* hydrocarbon biosynthesis that due to their hydrophobic properties [113], would contribute significantly to physical and biological protection, i.e. egg water retention and water balance. Indeed, *T*. *infestans* eggs were shown to synthesize hydrocarbons [114].

In the triatomines *Panstrongylus megistus and Dipetalogaster maxima*, lipid transfer to the developing oocyte during vitellogenesis is accomplished by endocytosis of Lp and by the classic extracellular Lp shuttle mechanism [28, 29]. However, in *R*. *prolixus*, endocytosis of Lp does not seem to be involved in lipid transfer to the oocytes [115, 116]. Interesting, in *D*. *melanogaster* the endocytic lipophorin receptor, LpR, promotes the extracellular hydrolysis of neutral lipids contained in lipoprotein particles, by an endocytosis-independent mechanism [117]. Here, our results show that *LpR* transcript levels are up-regulated in OV_FC. Thus, LpR in ovaries of *R*. *prolixus* could be working as an extracellular stabilization of Lp, promoting the extracellular lipolysis of Lp without endocytosing it. In addition, in mammals, it is known that once lipid levels drop, SREBP induces the expression of many genes involved in lipid synthesis and uptake, including the LDL receptor [118]. It has been reported that SREBP controls lipid uptake and accumulation in oocytes from *D*. *melanogaster* by regulation of LpR expression [119]. In our data we find up-regulation of *SREBP* mRNA in OV_FC (Fig 7 and S1 Table), suggesting that this transcription factor could be involved in lipid accumulation by the oocytes during vitellogenesis.

Diacylglycerol kinase (DGK) is a family of enzymes that catalyzes the conversion of diacylglycerol (DAG) to phosphatidic acid (PA). We find that *DGK* transcript expression is up-regulated in OV_FC (Fig 7 and S1 Table). PA is a component of the membrane phospholipids and at this stage there is a high demand for membrane synthesis, which is used for oocyte growth and/or for organelles formation, such as yolk granules and lipid droplets. On the other hand, PA affects numerous intracellular signaling pathways, including those regulating cell growth, differentiation, and membrane trafficking. Indeed, PA can bind to ToR and promote its signaling [120]. This finding further supports ToR signaling activation after a blood meal in OVs of *R*. *prolixus* [17]. Also, the requirement of *Rhopr*ACSL2 in fatty acid oxidation in the FB and promoting reproductive capacity in *R*. *prolixus* females has been reported [104]. Here we find that *RhoprACSL2* expression is higher than *RhoprACSL1* in both FB and OVs (Fig 7 and S1 Table), supporting the premise that *Rhopr*ACSL2 could have a more important role during the reproductive event in females of *R*. *prolixus*. Acyl-CoA-binding protein (ACBP) binds acyl-CoA esters with very high affinity to protect them from hydrolysis. Majerowicz et al., [121], reported that in *R*. *prolixus*, *Rp*ACBPs have characteristic expression profiles in different tissues, suggesting specific roles for each one. Although *RpACPB-2*, *RpACPB-3*, *RpACPB-4* and *RpACBP-5* transcripts are present in both tissues, only *Rp*-*ACPB-3* is up-regulated in FB_FC whereas *Rp*-*ACPB-4* is upregulated in OV_FC (Fig 7 and S1 Table). In comparing different tissues in *R*. *prolixus* females after feeding, Majerowicz et al., [121] found that RpACBP-2 and RpACBP-5 are expressed at high levels in the OV whereas RpACBP-3 and RpACBP-4 expression is equal in FB and OV. Here we find the expression of *RpACPB-2*, *RpACPB-3*, *RpACPB-4* and *RpACPB-5* is always higher in the OV than FB, with the highest level being *RpACPB-5* (Fig 7 and S1 Table). Interesting, knockdown of the transcript for *Rp*ACBP-5 has no effect on egg laying and hatching, or on accumulation of triacylglycerol in the oocytes. However, the authors do not rule out a key role of RpACBP-5 during reproduction and suggest that the result obtained by RNAi could be due to overlapping functions with the other proteins of the ACBP family, masking the potential role of RpACBP-5 on a successful reproductive event [122]. Overall, these finding indicate that the role of *Rp*ACPBs in lipid metabolism is specific for each tissue and physiological condition.

### Neuropeptides and neurohormonal signaling, and serotonin

**Fig 8:**
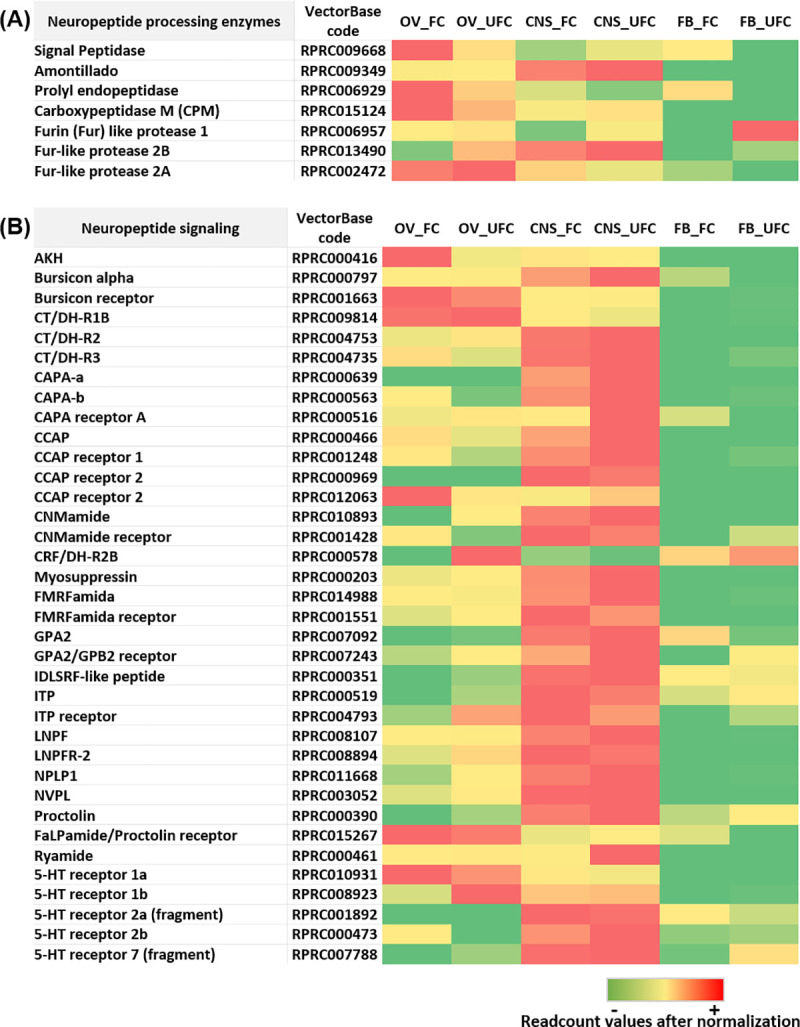
**Heat map comparing the mRNA expression levels of molecules involved in neuropeptide processing enzymes (A) and neuropeptide signaling (B) in fat body, ovaries and CNS of female adults in different nutritional condition.** The input data is the readcount value from the gene expression level analysis after normalization and is presented by means of a colour scale, in which green/yellow/red represent lowest/moderate/highest expression. DESeq was used to perform the analysis.

A variety of neuropeptides and neurohormones have been identified in the CNS of *R*. *prolixus* [123]. FB and OV development and function are largely regulated by several hormonal and nutritional signals, i.e. ILP/ToR signaling [17]. Our transcriptome analysis showed no significant change in mRNA expression after blood intake in CNS. However, we made a deep analysis in CNS, FB and OV to explore the relative expression of transcripts related to hormonal signaling in both nutritional conditions. Here, we discuss neuropeptides, in addition to the amine serotonin, and their receptors, which show high expression in some of the tissues analyzed (for more details see S1 Table). All neuropeptides are synthesized as part of a larger precursor molecule. The selective processing of those precursors determines which peptides are finally released by the specific cells [124]. Here, we find 7 enzymes involved in neuropeptide processing and all of them are expressed in the CNS, FB and OV in both nutritional conditions (Fig 8A and S1 Table). The results support the contribution of FB and OV for neuropeptide production in both nutritional condition.

The presence of the AKH precursor and its receptor in OVs suggests a role in egg production and/or egg-laying behaviour as has been shown in other insects [125, 126], possibly by an autocrine pathway. Here, we find that *AKH* transcript expression is detected in CNS but is up-regulated in OV_FC (Fig 8B and S1 Table). In insects, buriscon is a heterodimeric glycoprotein hormone which plays a key role in melanization and cuticle hardening during development of insects [127]. Recently, a novel function of bursicon was reported in the stimulation of Vg expression in the black tiger shrimp, *Penaeus monodon* [128]. In *R*. *prolixus*, we find higher expression of the *bursicon receptor* in OVs with respect to the CNS and FB (Fig 8B and S1 Table), suggesting a novel role for this hormone in reproductive physiology in an insect. Human genome screening reveals the presence of another glycoprotein hormone, consisting of the novel alpha (GPA2) and beta (GPB5) subunits (GPA2/GPB5) [129]. In *A*. *aegypti*, GPA2/GPB5 signaling has been implicated in controlling ionic balance [130]. In addition, this signaling pathway could play a role in spermatogenesis and oogenesis in male and female mosquitoes, respectively [131]. We find an up-regulation of *GPA2/GPB5 receptor* mRNA expression in OV and FB during UFC, suggesting an involvement of this signaling pathway in the stage prior to vitellogenesis (Fig 8B and S1 Table). Also, in rats, it has been reported that GPA2/GPB5 in the ovary may act as a paracrine regulator in reproductive processes [132]. Our results show up-regulation of *GPA2* mRNA in OV_UFC and conversely, up-regulation of this transcript in FB_FC (Fig 8B and S1 Table). Future experiments will determine the involvement of this new signaling pathway in insects and its interplay with reproductive processes. Calcitonin-like diuretic hormones (CT/DHs) are related to the mammalian calcitonin and calcitonin gene-related peptide hormonal system [133]. Here, in addition to the expression in CNS, we show a high mRNA expression level of *CT/DH-R*s in OV with moderate levels in the FB (Fig 8B and S1 Table). Previously, in *R*. *prolixus*, it was suggested that CT/DH-Rs signaling may have a critical, but unknown, role in reproductive physiology [134].

*R*. *prolixus* genome has two paralogue genes encoding capability (CAPA) peptides, named *Rhopr*CAPA*-α* and *Rhopr*CAPA*-β* [135, 136]. These genes are mainly expressed in the CNS, supporting our transcriptome results (Fig 8B and S1 Table). *Rhopr*CAPA-*α* expression was also detected in testes from 5th instar nymphs but not from adults, suggesting a role in the maturation of male gonads [135]. Here, we find *Rhopr*CAPA*-β* transcript expression up-regulated in OV_FC. Future experiments using gene silencing strategies will be performed to analyse the possible involvement of *Rhopr*CAPA*-β* peptides on oocyte maturation or egg formation.

Pleiotropic effects of crustacean cardioactive peptide (CCAP) in insects and crustaceans have been described [137]. Previously, it was reported that CCAP is involved in the fertilization process in *L*. *migratoria* since it increases the basal tonus and frequency of spontaneous spermathecal contractions [138]. Our results show an up-regulation of *CCAP* mRNA expression in OV_FC (Fig 8B and S1 Table), suggesting an autocrine regulation but future experiments are required to determine the specific involvement of this signaling in *R*. *prolixus* reproduction.

Ion transport peptides (ITPs) in locusts (*Schistocerca gregaria* and *L*. *migratoria*) were identified based on their antidiuretic activity on the ileum [139, 140]. In *T*. *castaneum* ITP signaling was involved in with the activation of the oviduct for egg laying [141]. Interestingly, in *B*. *mori* it was suggested that ITP signaling participates in regulating insulin and ecdysone signaling pathways [142]. However, its specific role in reproductive physiology in *R*. *prolixus* has not yet been reported. Here, we found an up-regulation of *ITP receptor* mRNA in both FB and OV from fed insects (Fig 8B and S1 Table).

In insects, long neuropeptide F (LNPF) has been reported as a main player in feeding behaviour, metabolism and stress responses [143]. Previously, in *R*. *prolixus*, it was reported that pre-follicular cells within the germarium express the NPF receptor (*Rhopr*NPFR), as do cells located between developing oocytes [144]. Also, it has been suggested that *Rhopr*NPF is capable of controlling aspects of reproduction since injection of *Rhopr*NPF results in an increase in the total number of eggs laid in *R*. *prolixus* [145]. Furthermore, NPF appears to be responsible for oocyte maturation and development in female locusts [146, 147]. Here, we find an up-regulation of mRNA expression of *Rhopr*NPFR in OV_UFC, a state where most oocytes are in an immature development stage and are maintained mainly by nurse cells located in germarium (Fig 8B and S1 Table). Therefore, we suggest that NPF signaling could also play a critical role in aspects of the physiology of the immature oocyte.

Neuropeptide-like precursor 1 (NPLP1) was first identified in *D*. *melanogaster* [148]. In *R*. *prolixus* NPLP1 peptides are involved in the feeding response, providing the first clues in the elucidation of their function [23]. We find an up-regulation of *NPLP1* transcript expression in OV_UFC (Fig 8B and S1 Table). The physiological role of NLPL1 signaling in reproduction is currently unknown.

By quantitative peptidomic assays, it was reported that in *R*. *prolixus*, NVP-like (NVPL) signaling is involved in the regulation of rapid events, such as diuresis/antidiuresis, and in delayed events such as mating and reproduction [23]. In our transcriptome analysis, we show an up-regulation of *NVPL* mRNA in OV_UFC (Fig 8B and S1 Table). Gene silencing techniques could be implemented to evaluate the role of this peptide in reproduction.

Myosuppressin is a neuropeptide only found in insects and crustaceans. It has been demonstrated to have anti-feeding activity and to inhibit gut and oviduct contraction and neuropeptide secretion [149]. In the Australian crayfish *Cherax quadricarinatus*, myosuppressin was detected in ovaries from mature females, suggesting a potential link between myosuppressin and reproduction [150]. Here, we also report the presence of *myosuppressin* mRNA in OVs of *R*. *prolixus* (Fig 8B and S1 Table).

A corticotropin-releasing factor-like peptide acts as a diuretic hormone in *R*. *prolixus* (Rhopr-CRF/DH) [151]; however, its distribution throughout the CNS and the expression of its receptor in feeding-related tissues as well as the female reproductive system suggests a multifaceted role for the neuropeptide. Adult female *R*. *prolixus*, injected with Rhopr-CRF/DH produce and lay significantly fewer eggs [152]. In addition, in locusts, CRF/DH inhibits oocyte growth and reduces ecdysteroid levels [153]. Here, we find an up-regulation of *CRF/DH receptor* mRNA in OV and FB from unfed insects (Fig 8B and S1 Table), where vitellogenesis is inhibited, supporting its effects as a negative regulator of reproduction.

By bioinformatic predictions, Ons et al. [154] showed for the first time the existence of RYamide in *R*. *prolixus*. However, the functions of this signaling in insects is currently unclear. We find a high expression of *RYamide* mRNA in OVs during both nutritional condition (Fig 8B and S1 Table).

Proctolin was the first insect neuropeptide to be sequenced and synthesized and is found in a variety of arthropods, including *R*. *prolixus* [155], where it plays a myostimulatory role on anterior midgut, hindgut, heart, and reproductive tissue [156]. In the cockroach *Blaberus craniifer*, nanomolar quantities of proctolin induce Vg uptake [157]. Here, we find for first time a high expression of *proctolin receptor* mRNA in OVs, encouraging further studies to analyze the role of this signaling in the reproductive organs (Fig 8B and S1 Table).

Serotonin (5-hydroxytryptamine or 5-HT) is an ancient monoamine neurotransmitter/neurohormone. 5-HT receptors are classified based on sequence similarities with their counterparts in vertebrates [158]. In *R*. *prolixus*, we find that mRNA expression to all *5-HT receptors* is higher in the CNS but also is expressed in the OV and FB (Fig 8B and S1 Table). In mosquitos, 5-HT2B was reported to be a critical player in the fat body-specific serotonin signaling system, governing the lipid deposition and ovarian development via ILP actions [159]. Also, it was shown that serotonin regulates an *Rp*ACBP-1 gene expression in the midgut of *R*. *prolixus*, reinforcing its role in lipid metabolism [160]. It would be interesting to analyse specific functional role to each 5-HT receptors in reproductive tissues of *R*. *prolixus*, mostly to link ILP signaling with serotonin.

The transcriptome data highlights directions for future research in examining the role of particular neuropeptides/amines on specific responses to processes such as ovarian maturation or egg formation, extending the temporal range of transcript/protein expression of these neuropeptides/amines capitalizing on gene silencing assays.

### A brief analysis of genes related to immunity

**Fig 9:**
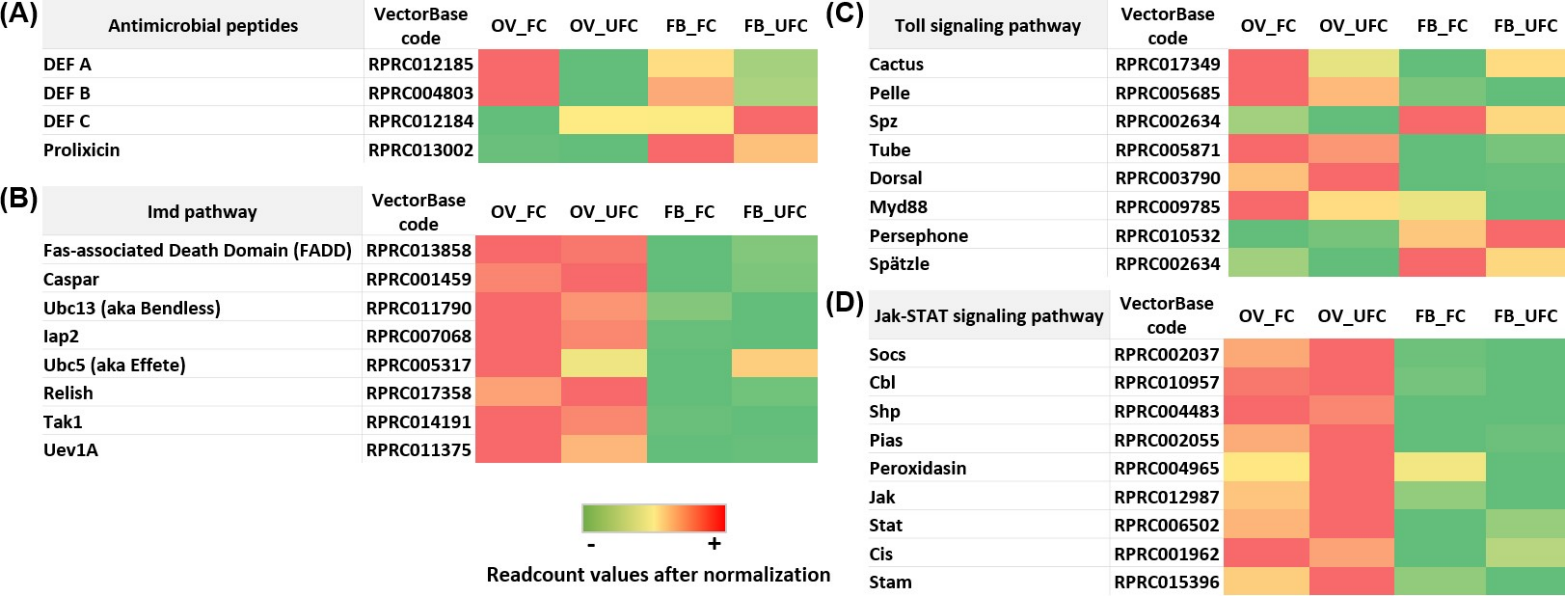
**Heat map comparing the mRNA expression levels of molecules involved with Antimicrobial peptides (A), Imd pathway (B), Toll signaling pathway (C) and Jak-STAT signaling pathway (D) in fat body and ovaries of female adults in different nutritional condition.** The input data is the readcount value from the gene expression level analysis after normalization and is presented by means of a colour scale, in which green/yellow/red represent lowest/moderate/highest expression. DESeq was used to perform the analysis.

The overall achievement of insects in maintaining a stable population of individuals is due, in part, to their ability to recognize pathogens and eliminate them successfully using the immune system. The immunity of insects comprises multiple elements that work in concert and, in general, includes physical barriers as well as innate immune responses, which lead to a combination of cellular and humoral immunity [161]. In recent years, it has been shown that reproduction and immunity can be mutually constraining since both responses are energetically costly, and therefore need to be traded off. In this context, increased reproductive activity reduces immunity across a diversity of female insects [162]. In addition, metabolic changes that occur after the acquisition of a blood meal promote the induction of oxidative stress [163]. Increased metabolic activity during the process of blood digestion has been shown to alter levels of different detoxification enzymes in mosquito, indeed blood feeding status in mosquitos confers increased tolerance to insecticides [164]. Thus, it is clear that in blood feeding insects, the immune system is working in both nutritional conditions, before a blood meal, due to the stress that is generated by starvation, and after a blood meal, due to the potential toxicity of the molecules ingested with the blood. Along with all the roles described above for FB in reproduction, this tissue also responds to microbial infection. One important humoral response is the production of inducible antimicrobial peptides (AMPs), which are rapidly synthesized after microorganism invasion [165]. In *D*. *melanogaster*, the Toll pathway (activated by fungi and gram-positive bacteria) and the Imd pathway (activated by gram-negative bacteria) lead to the synthesis of AMPs, not only by a pathogenic challenge, but also by aging, circadian rhythms, and mating [166–168]. It is noteworthy that several elements of the IMD pathway were reported as “missing” when the *R*. *prolixus* genome was published [16]. However, recently, Salcedo-Porras et al. [169] found orthologues for most of the “missing” elements of the IMD pathway in *R*. *prolixus* and reported that these are regulated in response to infection with Gram-negative bacteria. Interestingly, here we find an up-regulation of AMPs in OV_FC (Fig 9A and S2 Table), suggesting a role for humoral immunity in vitellogenic oocytes. In addition, we find several mRNAs involved with both, Toll and Imd pathways which are up- and down-regulated in FB and OV, without revealing a specific expression pattern in any of the nutritional conditions analyzed (Fig 9B and 9C and S2 Table). This finding may suggest that the immune system is responding to both stimuli: to detoxification of compounds which enter with blood intake and/or to avoid tissue damage due to stress caused by lack of food. In addition, FoxO transcriptional factor could promote activation of the stress-responsive Jun-N-terminal kinase (JNK) pathway, which antagonizes ILP signaling in *D*. *melanogaster*, causing nuclear localization of FoxO and inducing its targets, including growth control and stress defense genes [170]. Recently, we demonstrated that in unfed females, FoxO factor is translocated to the nucleus, stimulating the insulin-sensitive pathway and modulating longevity signaling in *R*. *prolixus* [17]. In the current work, we find up-regulation of most of the genes involved with JNK signaling, mainly in OV_UFC (Fig 9D and S2 Table) possibly to overcome effects of stress and low nutrition.

**Fig 10:**
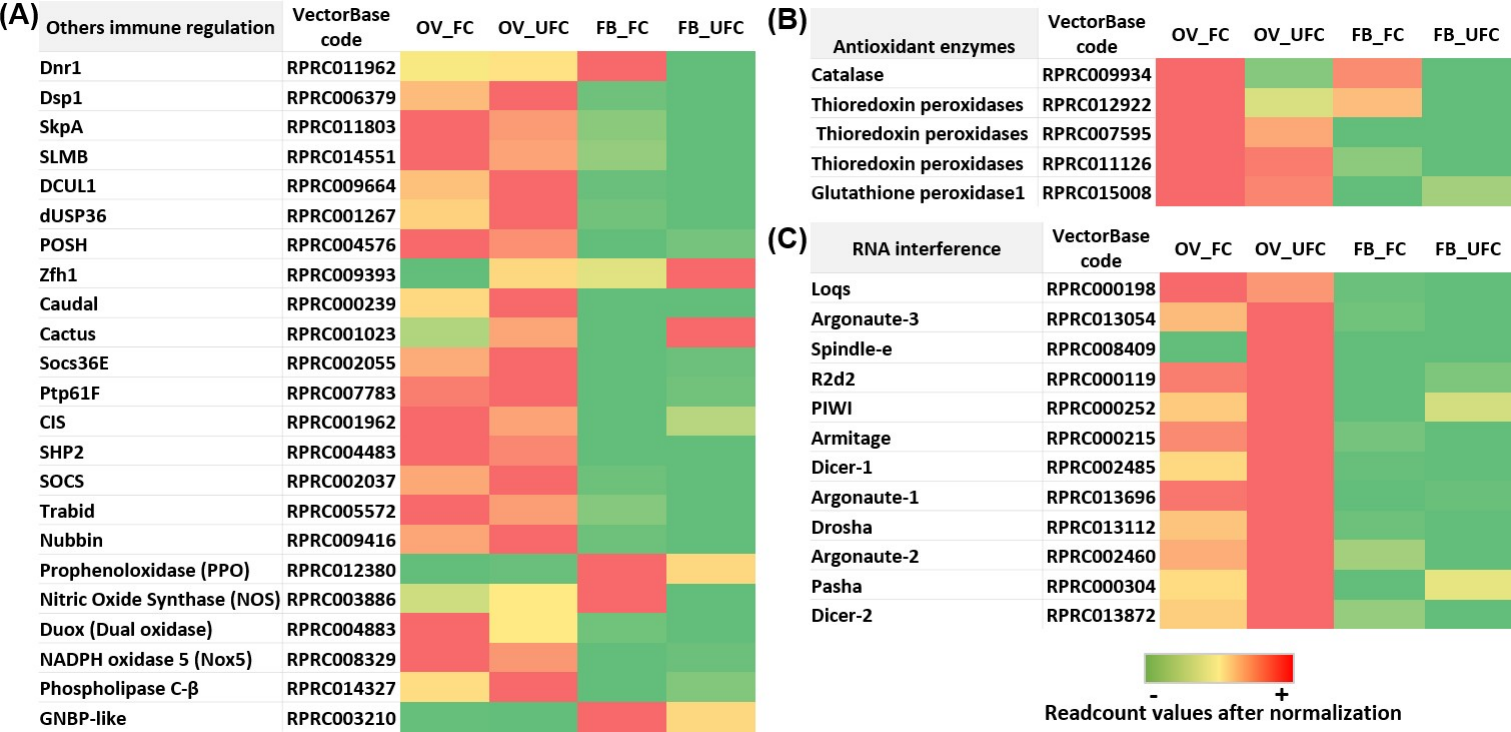
**Heat map comparing the mRNA expression levels of various immune regulators (A), antioxidant enzymes (B) and RNA interference signaling (C) in fat body and ovaries of female adults in different nutritional condition.** The input data is the readcount value from the gene expression level analysis after normalization and is presented by means of a colour scale, in which green/yellow/red represent lowest/moderate/highest expression. DESeq was used to perform the analysis.

In *R*. *prolixus*, Duox is the enzyme that generates H_2_O_2_ in ovarian follicles and it is used as a fuel for hardening of eggshell proteins, a process essential for the acquisition of resistance to water loss [171]. In accordance with those finding, we show an up-regulation of *Duox* mRNA expression in OV_FC (Fig 10A and S2 Table). In addition, melanization and the production of nitric oxide (NO) and reactive oxygen species (ROS) are effector mechanisms also activated as a first line of defense. Upon infection, pattern recognition receptors activate downstream serine protease cascades that culminate in the activation of prophenoloxidase (PPO), a precursor activated by proteolytic cascades to phenoloxidase for *de novo* synthesis of melanin. NO is highly toxic for a wide variety of pathogens and is produced by nitric oxide synthase (NOS). ROS are produced by conserved nicotinamide adenine dinucleotide phosphate (NADPH) oxidase enzymes [161, 165]. Recently, it was reported that L-arginine treatment in *R*. *prolixus* nymphs induced a higher NOS gene expression in the fat body and increased NO production in order to regulate the intestinal microbiota and control parasite development [172]. Here, we show the arginine biosynthesis pathway up-regulated in FB_FC along with an increase (not statistically significant) in NOS transcript expression, which could be regulating NO production in FB to control remotely the intestinal microbiota after a blood meal. Also, here we find up-regulation of *PPO* and *NOS* mRNA levels in OV_UFC (Fig 10A and S2 Table) but *PPO* up-regulated in FB_FC. In mammals, NO, produced by NOS [173], has emerged as one of several important intra-ovarian regulatory factors, including influencing steroidogenesis [174, 175]. In insects, cGMP signaling, co-regulated by NO, negatively controls ovarian steroidogenesis [176]. Supporting what other authors reported, we show that ovaries are able to synthesize ecdysteroids. In *R*. *prolixus*, the increased NOS in ovaries before a blood meal could be associated with a non-immune role, producing NO as an autocrine regulator of ovarian steroidogenesis. NADPH oxidase 5 (NOX5) present in mature follicles has been reported as essential for *D*. *melanogaster* ovulation [177]. Since NOX5 expression is higher in OV rather than FB, our results could indicate a non-immunity-related role of this enzyme on OV of *R*. *prolixus* females, signaling reproductive success, as was reported in *D*. *melanogaster*. Also, it is interesting to see that in general, enzymes which performs as antioxidant elements, such catalases and thioredoxin peroxidases have mRNA levels increased or up-regulated in OV and FB of fed insects (Fig 10B and S2 Table). RNA interference (RNAi) is triggered by endogenous or invading double-stranded RNAs (dsRNAs) that arise from hairpin structures, transposable elements, or virus infections [178]. In *R*. *prolixus* we show that in general, there is an up-regulation of mRNA molecules involved with RNAi signaling in OV_UFC (Fig 10C and S2 Table). These results suggest immunological signaling in OV of unfed insects, possibly to prevent damage during unfavorable metabolic conditions.

Overall, the information on immunity in hemipterans, including Triatominae vectors remains incomplete and fractionated [179]. The data presented here on immunity and reproduction in triatomine females encouraging the development of future studies to shed light on the relative contribution of the immune system in successful reproductive events.

## Conclusions

We present here a comprehensive analysis of mRNA expression of components of biological processes related with feeding and reproduction. Broadly, using high-throughput sequencing and a comparative expression analysis we find that a blood meal taken by *R*. *prolixus* females has both unique and interacting effects on CNS, OV and FB gene expression, with patterns of mRNA levels that are consistent with different needs according to the nutritional condition. Of particular interest, we show the cross-talk between reproduction and a) lipid, trehalose and protein metabolism, b) neuropeptide and neurohormonal signaling, and c) the immune system. Overall, our findings provide an invaluable molecular resource for future novel investigations on different tissues related with successful reproductive events, before and after the appropriated stimuli (blood meal). Our data opens up avenues of translational research that could generate novel strategies of vector population control. This includes, per example, the identification of specific genes for use in symbiont-mediated RNAi, a powerful technology which provides the potential for biocontrol against tropical disease vectors. In *R*. *prolixus*, the ability to constitutively deliver dsRNA by supplying with recombinant symbiotic bacteria generated against specific target genes involved in the reproductive success (Vg), have already been tested in laboratory trials and is effective in dramatically reducing the fitness of *R*. *prolixus* [18].

## Supporting information

S1 Fig Correlation of Log_2_Fold Change values in fat body (FB) and ovaries (OV) obtained by RNAseq and RT-qPCR data from 7 genes. Primers used are displayed in S3 Table. The correlation coefficient between RNAseq (y-axis) and RT-qPCR (x-axis) data (log_2_fold-change) analyzed by the Pearson test were 0.9311 (a) and 0.9109 (b), with a statistical significance p<0.01.
(TIF)

S2 Fig The evolutionary history of Vitellogenins from *Triatoma infestans* and *Rhodnius prolixus*. The evolutionary history was inferred by using the Maximum Likelihood method and JTT matrix-based model [181]. The tree with the highest log likelihood (-9963.68) is shown. The percentage of trees in which the associated taxa clustered together is shown next to the branches. Initial tree for the heuristic search were obtained automatically by applying Neighbor-Join and BioNJ algorithms to a matrix of pairwise distances estimated using the JTT model, and then selecting the topology with superior log likelihood value. There were a total of 1884 positions in the final dataset. Evolutionary analyses were conducted in MEGA X [182].
(TIF)

S1 Table Details of the mRNA expression of Figs 4 and 8. Columns are: the gene name we are assigning; VectorBase code–the official gene number in the RproC3 genome assembly; OV_FC, OV_UFC, FB_FC, FB_UFC, CNS_UFC and CNS_FC show the readcount after normalization. Log_2_FoldChange: log_*2*_ (fed condition/unfed condition); *p-*adj: *p*value after normalization (the smaller the *p-*adj, the more significant the difference). Excel cell highlights in green: up-regulation in fed condition; excel cell highlights in orange: up-regulation in unfed condition. CNS_FC, central nervous system post-feeding (FC, fed condition); CNS_UFC, central nervous system before of a blood meal (UFC, unfed condition); FB_FC, fat body in FC; FB_UFC, fat body in UFC; OV_FC, ovary in FC; OV_UFC, ovary in UFC.
(XLSX)

S2 Table Details of the mRNA expression of Figs 9 and 10. Columns are: the gene name we are assigning; VectorBase code–the official gene number in the RproC3 genome assembly; OV_FC, OV_UFC, FB_FC and FB_UFC are the readcount after normalization. Log_2_FoldChange: log_2_ (fed condition/unfed condition); *p-*adj: *p*value after normalization (the smaller the *p-*adj, the more significant the difference). Excel cell highlights in green: up-regulation in fed condition; excel cell highlights in orange: up-regulation in unfed condition.
(XLSX)

S3 Table Primers used by RT-qPCR assays. (XLSX)

S4 Table KEGG enrichment of pathways involved with amino acids metabolism and glycolysis and up-regulated after a blood meal in the fat body of *Rhodnius prolixus* females. The analysis was performed using KEGG database [180]. Statistical method: hypergeometric test [17].
(XLSX)

S5 Table KEGG enrichment of pathways involved with amino acids metabolism and glycolysis and up-regulated after a blood meal in the ovary of *Rhodnius prolixus* females. The analysis was performed using KEGG database [180]. Statistical method: hypergeometric test [17].
(XLSX)
