# Supplementary figures and images for: What happens after a blood meal? A transcriptome analysis of the main tissues involved in egg production in Rhodnius prolixus, an insect vector of Chagas disease
Source: PLoS Negl Trop Dis. 2020 Oct 15;14(10):e0008516. doi: 10.1371/journal.pntd.0008516 (PMC7591069; doi:10.1371/journal.pntd.0008516)

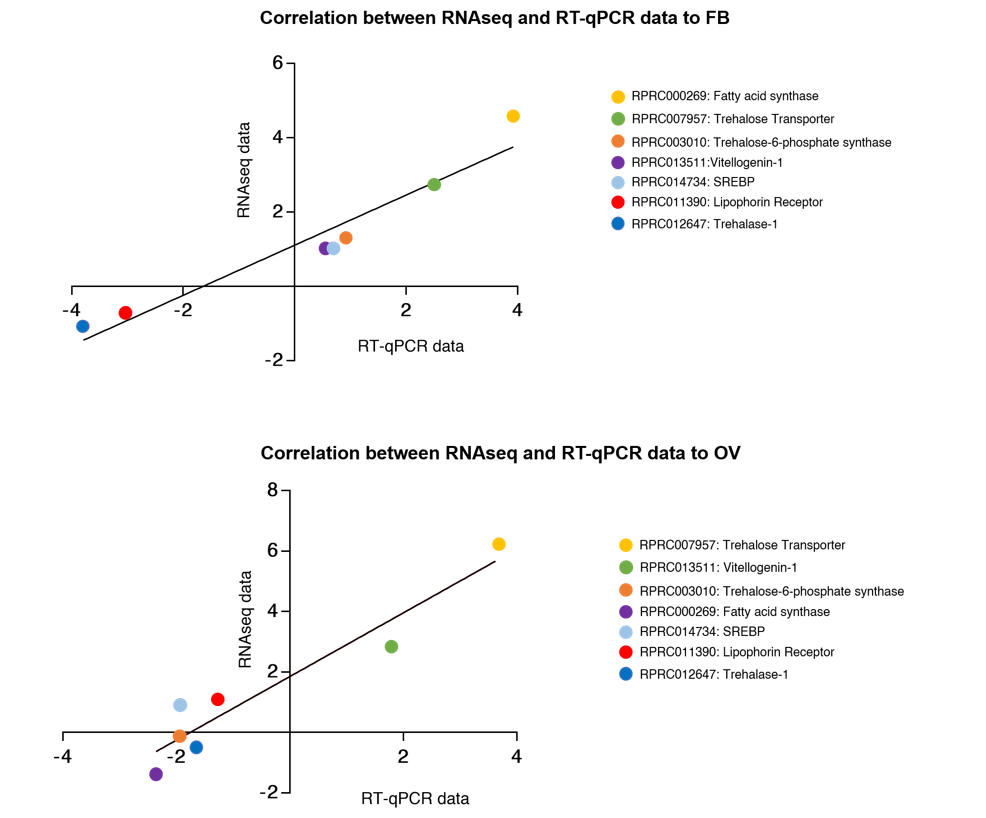

Supplement: S1 Fig — Primers used are displayed in S3 Table. The correlation coefficient between RNAseq (y-axis) and RT-qPCR (x-axis) data (log2fold-change) analyzed by the Pearson test were 0.9311 (a) and 0.9109 (b), with a statistical significance p<0.01. (TIF) [file pntd.0008516.s001.tif]

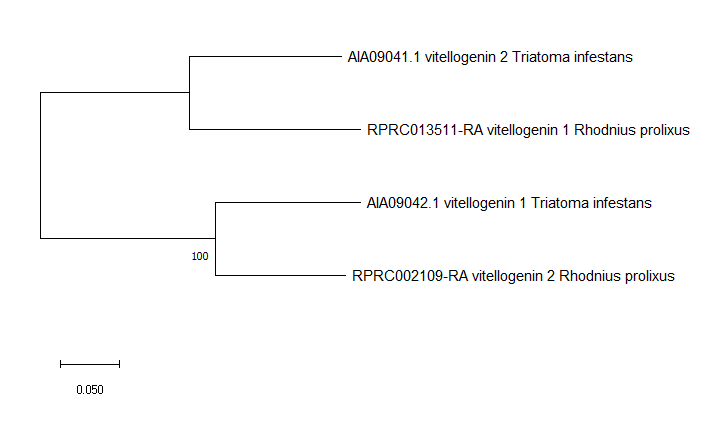

Supplement: S2 Fig — The evolutionary history was inferred by using the Maximum Likelihood method and JTT matrix-based model [181]. The tree with the highest log likelihood (-9963.68) is shown. The percentage of trees in which the associated taxa clustered together is shown next to the branches. Initial tree for the heuristic search were obtained automatically by applying Neighbor-Join and BioNJ algorithms to a matrix of pairwise distances estimated using the JTT model, and then selecting the topology with superior log likelihood value. There were a total of 1884 positions in the final dataset. Evolutionary analyses were conducted in MEGA X [182]. (TIF) [file pntd.0008516.s002.tif]
